# Point source capture with storage yields superior aviation health benefits over direct air capture

**DOI:** 10.1016/j.eehl.2026.100235

**Published:** 2026-03-21

**Authors:** Qiang Cui, Ying Zhou, Xing-yu Tang, Xu-jie Sun, Yu-xin Zhang, Ye Li

**Affiliations:** a School of Economics and Management, Southeast University, Nanjing 211189, China; b School of Business Administration, Nanjing University of Finance and Economics, Nanjing 210023, China

**Keywords:** Aviation emissions, Human health, CCUS, Airports

## Abstract

Emissions from the continuing expansion of the aviation sector present a serious threat to global climate and public health. Therefore, Carbon Capture, Utilization, and Storage (CCUS) technology—which comprises two main pathways, namely Carbon Capture and Utilization (CCU) and Carbon Capture and Storage (CCS)—has been investigated as a potential solution to mitigate aircraft emissions and reduce their associated public health impacts. CCUS capture approaches can be broadly categorized into two types: Direct Air Capture (DAC) and Point Source Capture (PSC). Only a limited number of studies assess the health impacts of emission reduction technologies. Exploring and comparing the health impacts of different pathways carries significant and far-reaching implications for sustainable development. Accordingly, in this paper, the Global Exposure Mortality Model (GEMM) is applied to assess the public health impacts of emissions from 1134 airports worldwide, covering approximately 94% of global operational airports. The findings reveal significant associations between aviation-related pollutants and number of deaths, particularly among men and older adults, with the Point Source Capture-Carbon Capture and Storage (PSC-CCS) pathway demonstrating the greatest potential for minimizing health risks. Then, a cost-benefit analysis shows that while all pathways yield negative net benefit—due to rising costs outpacing revenues—PSC-CCS remains the most economically viable option, with a maximum net benefit of -US$59.13 billion by 2050. In contrast, Direct Air Capture-Carbon Capture and Utilization (DAC-CCU) exhibits the poorest cost-effectiveness, limited by multiple technical and economic constraints.

## Highlights

• Four aviation CCUS pathways are compared based on mortality, and cost-benefit analysis.
• PSC-CCS yields the lowest mortality, with 2,074,699 deaths in 2050 compared to 4,824,776 for DAC-CCU.
• PSC-CCS shows the lowest net cost (-$59.13 billion by 2050).
• Prioritizing PSC-CCS cuts emissions and health risks.

## Graphical abstract

**Figure:**
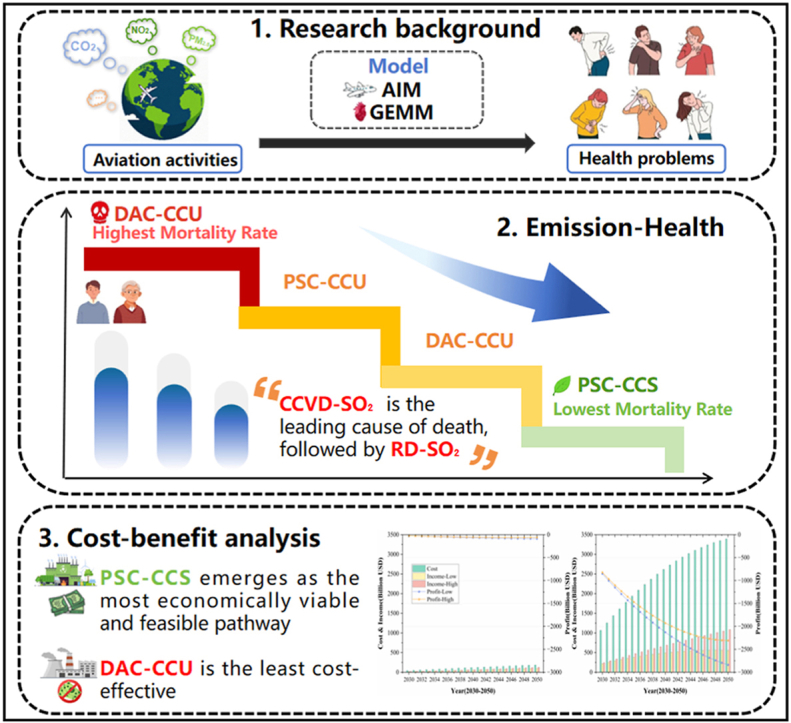

## 1 Introduction

The global civil aviation industry has experienced remarkable growth over the past few decades. In terms of passenger traffic, millions of passengers choose to travel by air each year, showing an upward trend. According to the International Air Transport Association (IATA), despite the impact of the COVID-19 pandemic in previous years, passenger volume had recovered to 4439 million by 2023. This momentum is attributed to the strong performance of the market and the sustained growth in international passenger volume. The Northeast Asia region is leading in passenger volume growth, while the strong demand from mature aviation markets such as Western Europe and North America has also made an important contribution to the industry’s overall growth [1]. In the coming years, supported by the continuous demand for air travel and the expansion of the aviation industry in major emerging economies, passenger volume is expected to continue to grow steadily.

The rapid growth in civil aviation demand is accompanied by substantial pollution emissions. As an important part of global economic activity, the impact of the aviation industry on climate change is mainly driven by the emission of CO_2_ from aircraft and other non-CO_2_ effects. The Intergovernmental Panel on Climate Change (IPCC) notes that since 1850–1900, the global surface temperature has risen by 1.1 °C, mainly due to human activities, especially greenhouse gas emissions [2]. The transportation sector is one of the industries with the highest carbon emission levels. From 2019 to 2022, transportation sector emissions were second only to those from the power and industrial sectors [3]. Among them, the impact of the aviation industry on global warming is significant. If no effective measures are taken, it is projected that by 2050 the aviation industry will cause a global temperature rise of 0.1 °C [4,5]. At present, many studies have examined the impact of civil aviation on climate change. These studies have quantified the contribution of civil aviation emissions to global warming, considering both CO_2_ and non-CO_2_ effects. The IPCC and other scientific institutions have emphasized the necessity of reducing emissions from the civil aviation industry to address the challenges of climate change. Due to the limited availability of climate mitigation strategies in the aviation industry—such as the use of low-carbon fuels, improved aircraft energy efficiency, and operational efficiency—and the lack of economically viable large-scale low-carbon strategies for aviation that can be widely promoted, as well as the higher demand growth of aviation compared to other modes of transportation, the aviation industry will face a key decarbonization challenge [6,7]. If significant emission-reduction measures are not taken, by 2100, the warming impact caused by the aviation industry is expected to increase significantly to 99.5 mK, equivalent to 5.2% of total human-induced warming [8]. Meanwhile, aviation-related PM also significantly impacts air quality and human health. In 2006, globally, aviation emissions led to an average PM_2.5_ concentration of 44.2 ng/m^3^ within a 20-km radius of all airports, and it is estimated that aviation emissions cause about 16,000 premature deaths annually [9].

To tackle the pollution emissions and climate change caused by the civil aviation industry, Direct Air Capture (DAC) within the broader Carbon Capture, Utilization, and Storage (CCUS) framework has garnered attention as a potential solution. CCUS technology serves as a vital instrument for achieving decarbonization and emissions reductions across various sectors. It represents one of the few technological options capable of maintaining the operation of existing coal-fired power plants (CFPPs) while significantly reducing their emissions [10]. The technology holds considerable potential for widespread adoption, supported by abundant onshore and offshore geological storage resources. When integrated with appropriate carbon pricing mechanisms and carbon markets, CCUS can facilitate the decarbonization of the power sector [11]. Furthermore, deep decarbonization of the cement industry is also expected to rely on CCUS. Prioritizing deployment in contexts with significant enhanced oil recovery (EOR) benefits can effectively lower the overall retrofitting costs in this sector [12]. However, some studies point out that under current carbon pricing and technological conditions, large-scale CCUS retrofitting in Chinese cement plants remains challenging in terms of feasibility [13]. Additionally, CCUS shows promising potential when coupled with other production technologies. For instance, coal-to-hydrogen coupled with Carbon Capture and Storage (C2HCCS) offers superior environmental benefits compared to conventional hydrogen production methods and remains more cost-competitive than hydrogen production via renewable energy-powered water electrolysis [14].

DAC-CCUS can remove CO_2_ from the atmosphere, offsetting the emissions from the aviation sector. There are six major technical approaches to remove CO_2_ and sequester: Coastal blue carbon, Terrestrial carbon removal and sequestration, Bioenergy with carbon capture and sequestration (BECCS), Carbon mineralization, Geological sequestration, and DAC [15]. When powered by renewable energy, direct air capture and storage is a promising method for the permanent removal of CO_2_ from the atmosphere [16]. Existing studies have examined the costs, feasibility, and environmental impacts of DAC and CCUS methods. Research indicates that, considering electricity costs and plant capacity factors, the cost of carbon capture and storage falls between €160/t CO_2_ and over €1300/t CO_2_ [17]. Another study suggests that while the initial costs of DAC are relatively high, they are expected to decrease significantly as technological deployment expands. The unit cost of DAC may initially be in the range of €500−€900/t CO_2_, but with large-scale deployment, this cost could be reduced to €90−€270/t CO_2_ [18]. Significant improvements in future innovation and learning could bring the projected average cost of deploying Direct Air Carbon Capture and Storage (DACCS) down by nearly a factor of five [17]. If commercialized in the 2020s, they could be implemented at scale by the 2040s and 2050s, when their scale might be comparable to existing climate mitigation achievements such as wind or solar energy [18]. Another factor affecting the feasibility of this technology is the huge energy demand. Capturing billions of tons of CO_2_ using DAC technology would require a massive amount of electricity, part of which might have to come from fossil fuels. The energy demand could reduce the carbon capture efficiency of DAC projects, put pressure on efforts to decarbonize the electricity supply, and limit the siting of DAC plants [19,20]. In terms of impacts, some research has pointed out that carbon capture and utilization would increase air pollution and total societal costs compared to not capturing carbon. If fossil fuel emissions exist, this situation cannot be changed by improving Carbon Capture and Utilization (CCU) equipment, because carbon capture always incurs equipment costs that wind energy never does, and adds to air pollution and fuel extraction [21]. The possibility of achieving negative emissions through CCS and DAC technologies is extremely low, as these technologies face numerous barriers such as high costs, technical infeasibility, and environmental concerns. Even if these technologies become less costly and more scalable in the future, they may not be able to meet the scale of negative emissions required to meet climate targets in a timely manner [22]. To address the problem of substantial energy demand, some researchers have conducted an in-depth exploration of DAC systems. One study proposed the integration of DAC systems with Heating, Ventilation, and Air Conditioning (HVAC) systems. By positioning the DAC unit downstream of the exhaust fan in an Air Handling Unit (AHU) [23], the system’s efficiency can be enhanced, with this energy-saving effect being most pronounced in cold climates [24]. Furthermore, another study analyzing two specific sorbents, Lewatit VP OC 1065 and SBA-15, found that SBA-15 holds a significant economic advantage over Lewatit [25]. Reducing greenhouse gas emissions at the source is of great importance.

Point source capture (PSC) combined with CCUS is another highly focused method. PSC-CCUS can directly capture CO_2_ from aircraft engines at airports or other concentrated emission sources. Current research has explored the possibility of integrating PSC-CCUS systems into airport infrastructure and the potential benefits in reducing emissions. Some studies have provided analytical and empirical insights into how PSC can be integrated with CCUS technologies and applied to the aviation sector to support emission reductions. Their research demonstrates that under the PSC-CCS pathway, CO_2_ is first captured via PSC from a fixed-point source, such as a waste-to-energy (WtE) plant, in an amount equivalent to the CO_2_ emitted during jet-fuel use in aviation. The captured CO_2_ is then transported to a designated storage site and permanently sequestered using CCS technology, thereby achieving negative emissions. This approach is not only cost-competitive but also considered technologically mature for widespread deployment [26].The most notable feature is that the PSC route costs less than the DAC route. This is because the low concentration of CO_2_ in the atmosphere means that DAC requires more energy. In addition, air transportation and sorbent regeneration in the DAC process also require energy. Theoretically, the minimum energy required for DAC is about 3.4 times that of point source capture [27]. At the same time, the energy consumption and CO_2_ emissions of the PSC route are significantly lower than those of the DAC route. The energy consumption of the PSC route is mainly related to the capture and transportation of CO_2_, whereas the DAC route requires a large amount of energy to capture CO_2_ from the atmosphere [28,29]. The PSC-CCS route is likely to remain the most cost-effective solution over the next few decades. Point-source capture mainly focuses on reducing CO_2_ emissions from the sources by separating CO_2_ from combustion flue gas through traditional capture technologies such as solvent-based absorption or cryogenic separation [30,31]. The advantage of this method is that it can directly reduce the CO_2_ emissions from specific emission sources. However, it usually requires a large amount of infrastructure investment and may be limited by the availability of suitable storage sites [32]. Point source CO_2_ capture and storage allow the continued use of fossil fuels in aircraft operations while largely eliminating their CO_2_ emissions. This technology can be implemented without significant changes for consumers, which helps address social inertia in climate mitigation efforts [33]. However, consumers still have to significantly change their demand for liquid hydrocarbon fuels [34].

The health impact of aviation emissions is also widely studied. Pollutants such as PM_2.5_, carbon monoxide (CO), nitrogen dioxide (NO_2_), and sulfur dioxide (SO_2_) emitted by aircraft engines can adversely affect human health. Studies have shown that exposure to these pollutants poses carcinogenic and non-carcinogenic risks, leading to respiratory and cardiovascular diseases, especially for populations living near airports [35]. Air pollutant particles smaller than PM_2.5_ can enter the respiratory system. Ambient air pollution may lead to chronic respiratory diseases, including chronic obstructive pulmonary disease, through inflammatory effects on the respiratory system [36,37]. On the other hand, it may also exacerbate hypertension and cause systemic inflammation and oxidative stress responses, thereby affecting various systems, including the cardiovascular system [38,39]. It may also lead to epilepsy through neuroinflammation, oxidative stress, and neuronal damage [40,41]. Aviation emissions have become an increasingly important source of ambient air pollution, with high-altitude emissions contributing as much as low-altitude emissions. The denser the aviation activity, the greater the health impact [42]. Meanwhile, populations living near airports are more severely affected by pollution. The closer one lives to the airport, the greater the risk of hospitalization for diseases such as asthma, chronic bronchitis, emphysema, and chronic obstructive pulmonary disease [43]. Flight routes over densely populated or industrial areas often accumulate a large amount of pollutants. Meteorological conditions—such as high summer temperatures and stable atmospheric conditions that lead to the formation of inversion layers—and the number of flight routes can also contribute to higher PM_2.5_ concentrations [44]. Among other emissions, CO is a common component of indoor and outdoor air pollution, mainly from incomplete combustion. CO can bind with hemoglobin, reducing the blood’s oxygen-carrying capacity, leading to hypoxia in the human body, which can be fatal in severe cases. Even low concentrations of CO can affect neurodevelopment, with particularly serious effects on infants and children [45]. Previous studies have found a robust association between SO_2_ and chronic obstructive pulmonary disease and cardiovascular diseases. NO_2_ has a robust impact on childhood asthma, premature birth, lung cancer, and diabetes [46]. SO_2_ has a strong, irritating odor that can damage the human respiratory system, causing symptoms such as coughing and wheezing, triggering respiratory tract irritation and inflammation, and increasing the risk of chronic respiratory diseases [47]. It also significantly contributes to deaths from cardiovascular and respiratory diseases [48]. Short-term exposure to NO_2_ can cause respiratory tract inflammation. At high concentrations, healthy individuals may suffer severe lung damage, and people with chronic respiratory diseases (such as asthma) may experience sudden respiratory reactions. Long-term exposure is associated with an increase in respiratory symptoms, with asthma patients, young children, and the elderly being more sensitive to its effects [49,50]. In summary, the impact of pollutants emitted by aircraft on human health is extensive and far-reaching. Effective environmental policies and technological improvements are needed to reduce the harm of these emissions to public health. Emissions from multiple pollutants in sea and air transport pose a significant threat to public health, necessitating the development of effective exposure reduction strategies. Research has extensively explored the health impacts of various air pollutants. Exposure to fine particles and gaseous pollutants (PM_10_, PM_2.5_, SO_2_, and NO_2_) contributed to a higher mutation rate in Mycobacterium tuberculosis [51], resulting in the development of active pulmonary tuberculosis [52]. Exposure to a mixture of air pollutants—including PM_2.5_, SO_2_, NO_2_, and CO—is detrimental to cardiovascular health [53]. Long-term exposure to a multipollutant mixture containing ozone, PM_2.5_, and NO_2_ significantly increases the prevalence of cardiovascular disease [54]. Furthermore, long-term individual and combined exposure to a mixture of PM_2.5_, SO_2_, and NO_2_ shows a robust positive association with mortality from cardiovascular diseases, non-malignant respiratory diseases, and lung cancer, with PM_2.5_ contributing the largest proportion of risk [55].

In summary, the existing literature has provided relatively comprehensive analyses of the emission reduction performance, application processes, and costs of DAC, PSC, and CCUS, and has also touched upon the climate impacts and health damages associated with air pollutants. Only a limited number of studies integrated these two aspects to assess the health impacts of these emission-reduction technologies. Aviation emissions contribute not only to global warming but also pose significant public health risks, a critical issue that warrants global attention. Exploring and comparing the health impacts of different technological pathways for reducing aviation emissions carries significant and far-reaching implications for the sustainable development of human society. Therefore, this paper establishes four scenarios: DAC-CCS, DAC-CCU, PSC-CCS, and PSC-CCU. Based on the technical framework of these scenarios, it calculates the emissions of each scenario in each cycle (see details in Supplementary Sections S5 and S7) and examines the public health impacts of the aviation industry across different pathways. These four scenarios differ in their economic, environmental, and social impacts [56,57]. Through these scenario analyses, this study evaluates their implications for the health of residents living near airports. In addition, a cost-benefit analysis is conducted to identify the most cost-effective emission reduction strategies. Overall, this paper aims to provide scientifically grounded emission reduction pathways for the aviation industry to protect public health. Furthermore, the findings will serve as a basis for policymakers in formulating more effective environmental policies and aviation development plans.

## 2 Material and method

### 2.1 Scenario setting

Because DAC and PSC projects have not yet been officially implemented in the aviation industry, this study assumes that their current usage is zero. Four scenarios are established: the DAC-CCS scenario, the DAC-CCU scenario, the PSC-CCS scenario, and the PSC-CCU scenario. To better evaluate the potential effects of CCUS technologies in aviation, the Business as Usual (BAU) scenario in this study assumes that clean technologies such as hydrogen energy have already been adopted. Therefore, the calculation results of the H2_Mid scenario from the Aviation Integrated Model (AIM) [58] are used as the BAU scenario outcomes. A brief introduction to the AIM model can be found in Section S1.

For the two CCS scenarios, it is assumed that carbon emissions under the BAU scenario can be completely offset by 2050. For the two CCU scenarios, it is assumed that the fossil energy used under the BAU scenario will be fully replaced by 2050. In addition, this study assumes that the annual usage ratios of CCS and CCU technologies change proportionally over time. Detailed descriptions of the CCS and CCU technology pathways are provided in Sections S4 and S6. All results are presented as differences between the four scenarios and the BAU scenario.

The DAC-CCS pathway first requires the construction of DAC plants at suitable locations to capture CO_2_ from the air. The amount of CO_2_ captured is equivalent to aviation emissions, thereby indirectly enabling emission reductions for the aviation sector. The captured CO_2_ is then purified and dehydrated before being transported via pipeline or tanker to storage sites. The DAC-CCU pathway is initially similar to DAC-CCS, involving the establishment of DAC plants to capture an equivalent amount of CO_2_ from the air. Subsequently, the DAC-captured CO_2_ is converted into intermediate products, which, together with H_2_, are fed into a Fischer-Tropsch synthesis system to produce synthetic aviation fuel and other products for use by the aviation industry. The PSC-CCS pathway first involves identifying the point source for PSC capture, which in this study is a WtE plant. Capture units are installed at locations such as the plant’s flue stacks to conduct CO_2_ capture. After capturing an amount of CO_2_ equivalent to aviation emissions at the WtE plant, the CO_2_ is processed and transported via pipeline or tanker to storage sites. Finally, the PSC-CCU pathway first uses PSC technology to capture CO_2_ from the WtE plant. It then utilizes CCU technology to convert a portion of this CO_2_ into intermediate products, which are combined with H_2_ to synthesize fuels useable by the aviation industry, while the remaining portion is sequestered.

### 2.2 Global Exposure Mortality Model (GEMM)

According to existing research [44], PM_2.5_ is associated with six diseases: (1) stroke; (2) lung cancer; (3) lower respiratory infections; (4) chronic obstructive pulmonary disease; (5) ischemic heart disease; and (6) type 2 diabetes. CO is linked to cardiovascular diseases, SO_2_ affects both respiratory and cardiovascular diseases, and NO_2_ impacts respiratory diseases [35].

In this study, the Gaussian diffusion model [59] is first used to calculate the average concentrations of PM_2.5_, CO, SO_2_, and NO_2_ caused by aircraft activities at each airport based on emission data. Subsequently, the GEMM [44], together with relevant population and disease data, is employed to estimate the impacts of these pollutants emitted by aircraft activities at 1134 airports worldwide on the mortality rates of residents living within 20 km of each airport from 2025 to 2050. The baseline mortality rates for various diseases are obtained from the Global Burden of Disease dataset [60], and population data within 20 km of each airport are sourced from LandScan [61]. For a detailed introduction to the Gaussian diffusion model and the GEMM, see Section S2.

### 2.3 Cost-benefit analysis method

The cost of alternative energy adoption mainly consists of the cost difference between alternative energy and JET-A. JET-A refers to a standard type of kerosene-based aviation turbine fuel. Revenues are primarily derived from the carbon price multiplied by the carbon emissions saved. That is

(1) $$C=C_{AE}-C_{JF}$$

(2) $$C_{AE}=N_{A}\times P_{A}$$

(3) $$C_{JE}=N_{J}\times P_{J}$$

Where, C denotes the cost of alternative energy adoption, defined as the difference between cost of the alternative energy and JET-A, expressed in US dollars (USD); $C_{AE}$ and $C_{JF}$ are the cost of alternative energy and jet fuel, reported in USD; $N_{A}$ (t) and $P_{A}$ (USD/t) are the number and price of alternative energy, and $N_{J}$ (t) and $P_{J}$ (USD/t) are the number and price of jet fuel.

(4) $$Re={SN}_{C}\times P_{C}$$

(5) N=Re−C

Where, Re (USD) means the revenues of alternative energy adoption; ${SN}_{C}$ (t) and $P_{C}$ (USD/t) are the number and price of carbon. The ${SN}_{C}$ is the carbon emission difference between the BAU scenario (the H2_Mid scenario of the AIM model) and the four CCUS scenarios (DAC-CCS, DAC-CCU, PSC-CCS, and PSC-CCU). *N* (USD) is the net benefit of alternative energy adoption.

According to existing research [26], in 2020, the cost per ton of fuel for DAC-CCS was approximately $2649.90, while for DAC-CCU it was around $7549.90. Forecasts indicate that by 2050, the cost per ton of fuel for DAC-CCS will decrease to approximately $1144.30, while that for DAC-CCU will decline to around $4062.50. In 2020, the cost per ton of fuel for PSC-CCS was approximately $856.65, compared to $4283.25 for PSC-CCU. By 2050, the cost for PSC-CCS is projected to decrease to about $697.00 per ton of fuel, while the cost for PSC-CCU is expected to decline to approximately $3252.88.

The forecasted prices of JET-A fuel and carbon from 2024 to 2050 are provided in Section S3.

We did not directly include the costs of CO_2_ transport, storage, or utilization as explicit items in our calculations. Instead, these costs are indirectly reflected in the price of alternative energy, which represents the final market price and thereby captures the costs incurred throughout the entire process. Similarly, our approach indirectly incorporates the economic benefit into our cost-benefit analysis by offsetting the airline’s need to purchase additional conventional fuel.

### 2.4 Data and code availability

The average annual wind speed at each of the 1134 airports is presented in Dataset S1. This parameter is essential for the Gaussian diffusion model and is used to calculate emission concentrations.

Dataset S2 provides the distribution of the global population living within 20 km of each of the 1134 airports, and Dataset S3 contains the concentration values of pollutants caused by aircraft emissions near each airport under the four scenarios. These two datasets are used to estimate the mortality rates attributable to aircraft activities for residents living near these airports.

The emission data for the BAU scenario used in this study are derived from the projection results of the AIM model (H2_Mid scenario).

The price data employed in the cost-benefit analysis can be found in the Section S3.

### 2.5 Sensitivity analysis

To assess the robustness of the results, this study conducts sensitivity analyses on both the mortality forecasts under the four technological pathways and the cost of CCUS. Detailed results are provided in Section S8-S9.

## 3 Results

### 3.1 Premature deaths attributable to aviation emissions

**Fig. 1:**
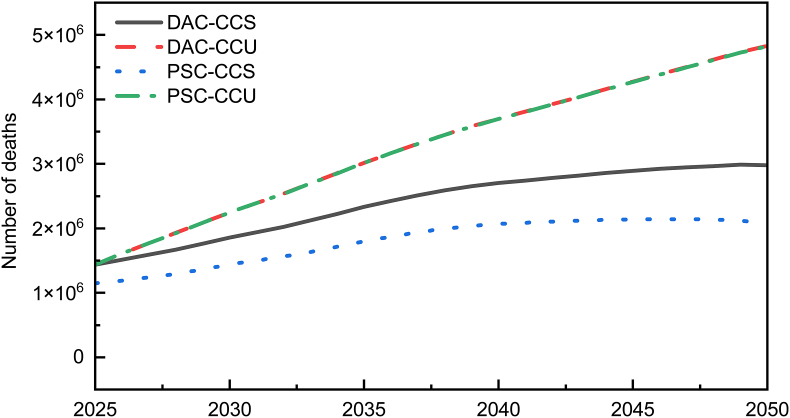
Mortality attributable to aviation emissions under four scenarios from 2025 to 2050. PSC, Point Source Capture; DAC, Direct Air Capture; CCS, Carbon Capture and Storage; CCU, Carbon Capture and Utilization.

Premature deaths attributable to aviation emissions vary significantly over time, as shown in Fig. 1. Overall, the number of premature deaths associated with the four scenarios increased gradually from 2025 to 2050. Among them, the PSC-CCS scenario exhibited the lowest health impact, with a decline beginning in 2046. The DAC-CCS scenario had the second-lowest health impact, with a decline starting in 2049. The other two scenarios, PSC-CCU and DAC-CCU, showed highly overlapping trends in health impacts between 2025 and 2050, with no significant differences. Moreover, these two scenarios had the highest health impacts. Specifically, in 2050, the DAC-CCU scenario had the highest number of premature deaths, at 4,831,322, followed by the PSC-CCU scenario with 4,824,776 deaths, while the PSC-CCS scenario had the lowest number of premature deaths, at 2,074,699. These results indicate that the PSC-CCS method has the lowest health impact on the population.

### 3.2 Health effects of aviation emissions on different genders

**Fig. 2:**
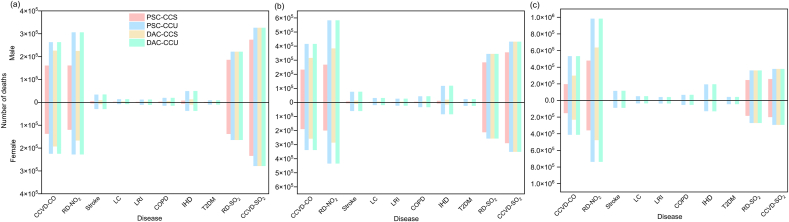
Gender-specific mortality attributable to aviation emissions under four scenarios in 2030 (a), 2040 (b), and 2050 (c). CCVD-CO, cardiovascular and cerebrovascular disease caused by CO; RD-NO_2_, respiratory disease caused by NO_2_; LC, lung cancer; LRI, lower respiratory infections; COPD, chronic obstructive pulmonary disease; IHD, ischemic heart disease; T2DM, type 2 diabetes mellitus; CCVD-SO_2_, cardiovascular and cerebrovascular disease caused by SO_2_; RD-SO_2_, respiratory disease caused by SO_2_.

Aviation emissions have differential health impacts on males and females. Fig. 2 illustrates the gender-specific mortality impacts of pollutants in 2030, 2040, and 2050. It is evident that males have a higher mortality rate due to pollution exposure than females.

Among the four scenarios, the diseases with the largest gender differences in mortality are RD-NO_2_ (respiratory disease caused by NO_2_) and RD-SO_2_ (respiratory disease caused by SO_2_), and this disparity increases over time. In 2050, under the DAC-CCU scenario, RD-NO_2_ results in 246,770 more male deaths than female deaths, while RD-SO_2_ leads to 90,197 more male deaths than female deaths. The gender difference in mortality related to cardiovascular and cerebrovascular disease (CCVD) is also relatively pronounced, which may be attributed to physiological differences between males and females. Notably, for type 2 diabetes mellitus (T2DM), the mortality rate among females exposed to pollutants is slightly higher than that of males across all scenarios. In 2050, under the DAC-CCU scenario, T2DM results in 1814 more female deaths than male deaths.

### 3.3 Health effects of aviation emissions on different age groups

**Fig. 3:**
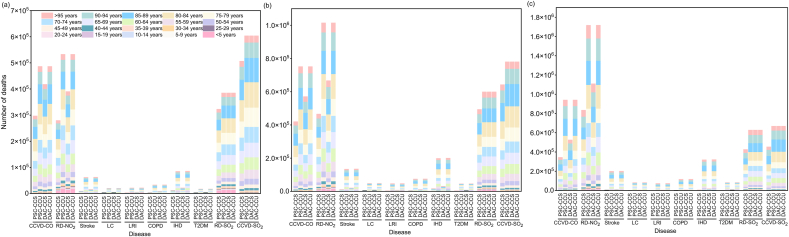
Age-specific mortality attributable to aviation emissions under four scenarios in 2030 (a), 2040 (b), and 2050 (c).

Fig. 3 presents the mortality impacts of aviation emissions on different age groups in 2030, 2040, and 2050. The data show that the influence of emissions on various age groups has changed over time. In 2030, across all four scenarios, the number of deaths increased with age, ranging from the lowest in the 5−9 years group to the highest in the 80−84 years group. However, for those aged 84 and above, deaths decreased with increasing age. By 2040 and 2050, the age group with the highest mortality due to pollutants shifted to 85−89 years, while the 5−9 years group still had the lowest mortality. In 2050, under the DAC-CCU scenario, 797,016 individuals aged 85−89 died from exposure to pollutants. Notably, mortality rates in the 70−74 years and 75−79 years age groups declined significantly between 2040 and 2050. Among the four scenarios, the PSC-CCS scenario consistently had the lowest mortality rates across all age groups, while the PSC-CCU and DAC-CCU scenarios had the highest.

In terms of specific diseases, in 2030, cardiovascular and cerebrovascular disease caused by SO_2_ (CCVD-SO_2_) was the leading cause of death among the elderly. Under the DAC-CCU scenario, 97,056 individuals aged 80–84 died from this disease. By 2050, RD-NO_2_ became the most fatal disease for the elderly. Under the DAC-CCU scenario, 250,702 individuals aged 85–89 died from this disease. In contrast, among the 0–14 years age group, mortality from lung cancer (LC), chronic obstructive pulmonary disease (COPD), ischemic heart disease (IHD), and T2DM due to PM_2.5_ exposure was very low, with these diseases having the lowest number of deaths in this age group.

The impact of pollutants on the working-age population (20−44 years) should not be overlooked. In 2050, under the DAC-CCU scenario, RD-SO_2_ was the most fatal disease in the 20−44 years age group, resulting in 52,755 deaths. The total mortality in this age group under this scenario reached 123,685. Even under the PSC-CCS scenario, which had the lowest mortality, the 20−44 years age group still recorded 56,089 deaths.

### 3.4 Health effects of aviation emissions on different diseases

**Fig. 4:**
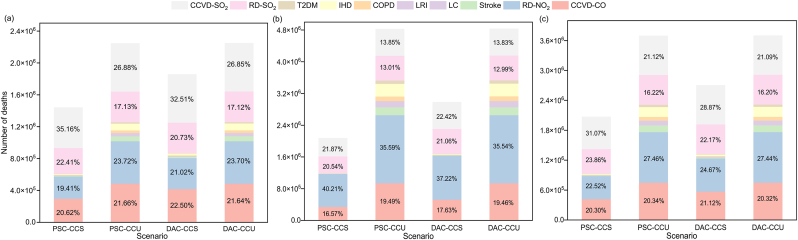
Disease-specific mortality attributable to aviation emissions under four scenarios in 2030 (a), 2040 (b), and 2050 (c).

Fig. 4 illustrates the mortality and percentage distribution of 10 diseases caused by aviation pollutants across the four scenarios in 2030, 2040, and 2050. Overall, the highest mortality rates are attributed to CCVD-SO_2_, RD-SO_2_, RD-NO_2_, and CCVD-CO. Firstly, cardiovascular and cerebrovascular disease and respiratory disease are significant causes of global mortality. Secondly, the toxic effects of NO_2_, SO_2_, and CO exacerbate inflammatory responses. CO binds with hemoglobin, reducing oxygen transport and causing tissue hypoxia, which can damage myocardial function.

In 2030, under the PSC-CCS scenario, the number of deaths due to CCVD-SO_2_ was 506,493, accounting for 35.16% of all disease-related deaths. By 2050, under the DAC-CCU scenario, the number of deaths due to RD-NO_2_ reached 1,717,220, representing 35.54% of all disease-related deaths. In 2030, CCVD-SO_2_ had the highest mortality proportion among all diseases. However, over time, the disease with the highest mortality shifted to RD-NO_2_ in 2040 and 2050. In contrast, the mortality rates of the six diseases caused by PM_2.5_ exposure were relatively low and close to zero.

### 3.5 Health effects of aviation emissions on different airports

**Fig. 5:**
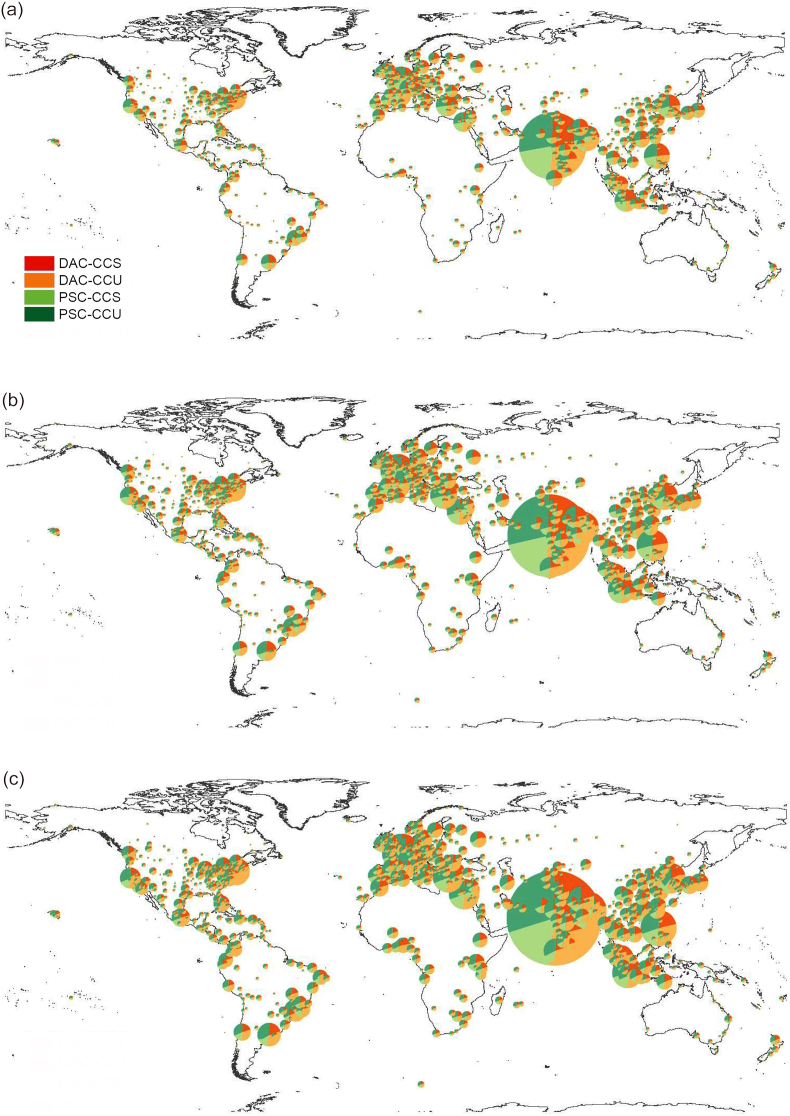
Mortality attributable to aviation emissions by airport under four scenarios in 2030 (a), 2040 (b), and 2050 (c).

Fig. 5 shows the number of deaths at 1134 airports worldwide under four scenarios in 2030, 2040, and 2050. Overall, the number of deaths at various airports illustrates an upward trend over the years. The number of deaths caused by pollutants at airports using CCS technology is generally lower than that at airports using CCU technology. Meanwhile, PSC-based pathways result in smaller health impacts on people near the 1134 airports worldwide, whereas the impact of pollutants on health is the greatest in the DAC-CCU scenario. Generally, the airports with the highest death toll include Chhatrapati Shivaji Maharaj International Airport, Paris Charles de Gaulle Airport, Netaji Subhas Chandra Bose International Airport, Ninoy Aquino International Airport, Incheon International Airport, Josep Tarradellas Barcelona-El Prat Airpor, Soekarno-Hatta International Airport, Athens Eleftherios Venizelos Airport, Indira Gandhi International Airport, Cairo International Airport, and John F. Kennedy International Airport. Among them, the number of deaths caused by pollutants emitted from Chhatrapati Shivaji Maharaj International Airport in India was the largest. In the DAC-CCU scenario in 2050, 533,769 people died. The number of deaths caused by RD-NO_2_ is the highest, reaching 111,400. Secondly, there is Paris Charles de Gaulle Airport in France. The death toll near the airport is 91,558. In 2030, the top 10 airports with the highest mortality were completely consistent across the four technological pathways, showing the same trend. By 2040, however, Cairo International Airport entered the list of the top 10 airports with the highest mortality, while John F. Kennedy International Airport dropped out. Meanwhile, under the two pathways applying CCU technology, Athens Eleftherios Venizelos Airport and Cairo International Airport reported relatively higher mortality; correspondingly, under the two CCS-based pathways, Soekarno-Hatta International Airport and Indira Gandhi International Airport showed higher mortality figures. By 2050, disparities among the four pathways became more pronounced. Overall, mortality near Cairo International Airport increased substantially, while the rankings of Athens Eleftherios Venizelos Airport and Josep Tarradellas Barcelona-El Prat Airport declined relatively. Across all three years and under all four scenarios, four airports consistently remained among those with the highest global mortality: Chhatrapati Shivaji Maharaj International Airport, Paris Charles de Gaulle Airport, Netaji Subhas Chandra Bose International Airport, and Ninoy Aquino International Airport.

### 3.6 Cost-benefit analysis of different DAC and PSC scenarios

**Fig. 6:**
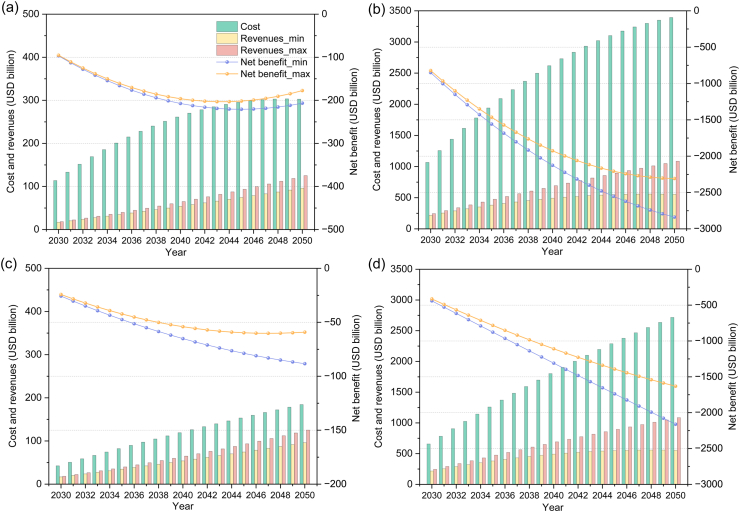
Cost-benefit analysis of the four scenarios: (a) DAC-CCS, (b) DAC-CCU, (c) PSC-CCS, and (d) PSC-CCU during 2030−2050. Revenues_min and Revenues_max denote the minimum and maximum estimated revenues, respectively; Net benefit_min and Net benefit_max denote the corresponding estimates for net benefit.

The costs and revenues of the four DAC and PSC scenarios are illustrated in Fig. 6, which shows the economic performance of each scenario. In 2030, DAC-CCS incurs a cost of $113.65 billion, with a net benefit ranging from -$96.89 billion to -$95.37 billion. Over time, both costs and revenues increase, but the cost growth consistently outpaces revenue growth, leading to a continuous decline in net benefit until 2043. By 2040, DAC-CCS exhibits a net benefit range of -$207.33 billion to -$196.29 billion. Starting in 2043, the net benefit begins to improve, with the maximum negative value reaching its lowest point at -$203.21 billion that year. The minimum net benefit hits its lowest point of -$220.89 billion in 2045 and then begins to rise as well. By 2050, the net benefit of DAC-CCS improves to a range of -$206.67 billion to -$177.44 billion. In contrast, DAC-CCU demonstrates significantly poorer economic performance, driven by a substantial increase in additional costs. In 2030, the net benefit ranges from -$849.83 billion to -$822.31 billion. Although revenues grow compared to DAC-CCS, the increase is far outpaced by the growth in costs. By 2040, DAC-CCU’s net benefit falls between -$2126.18 billion and -$1925.68 billion, and continues to deteriorate over the next decade. By 2050, the range further worsens to -$2838.11 billion to -$2307.40 billion, with the gap between maximum and minimum values also widening. Overall, As shown by Fig. 6, the cost of DAC-CCU is substantially higher than that of DAC-CCS, leading to marked differences in net benefits. Specifically, the maximum cost of DAC-CCS is close to $300 billion, whereas the maximum cost of DAC-CCU reaches nearly $3500 billion. Therefore, DAC-CCU’s extreme costs result in lower net benefit performance compared to DAC-CCS.

Between the two PSC pathways, while some patterns mirror those of DAC, there are also distinct differences. In 2030, the net benefit of PSC-CCS ranges from -$25.75 billion to -$24.23 billion. Over time, both costs and revenues rise steadily, but costs remain consistently higher than revenues. By 2040, the net benefit declines to a range between -$65.14 billion and -$54.09 billion. In 2047, the maximum net benefit value reaches its lowest point at -$60.23 billion, after which it begins to improve. By 2050, the upper bound of net benefit improves slightly to -$59.13 billion, while the full range extends from -$88.37 billion to -$59.13 billion. Meanwhile, the minimum net benefit continues to decline throughout the period. Compared to PSC-CCS, PSC-CCU shows a much steeper deterioration in net benefit, along with a significantly faster rise in costs. In 2030, the net benefit of PSC-CCU ranges from -$440.98 billion to -$413.47 billion. By 2040, it worsens to a range of -$1310.05 billion to -$1109.56 billion. In 2050, both the maximum and minimum net benefit values reach their lowest levels, ranging from -$2161.90 billion to -$1631.19 billion. Similar to the DAC case, the net benefit of PSC-CCU is substantially lower than that of PSC-CCS. Although both costs and revenues increase over time, the revenues remain consistently lower than the costs. Both pathways maintain negative net benefit values throughout the period, but PSC-CCS shows a relatively stable decline, while PSC-CCU experiences more rapid deterioration and starts with significantly higher losses.

## 4 Discussion

This study investigates the environmental, health, and economic performance of four CCUS technology application scenarios in the aviation industry. The GEMM model is applied to assess the health impacts of aircraft emissions—PM_2.5_, CO, SO_2_, and NO_2_—on residents living near 1134 airports worldwide from 2025 to 2050. Finally, a cost-benefit analysis is conducted for the four carbon capture pathways—DAC-CCS, DAC-CCU, PSC-CCS, and PSC-CCU—during the period from 2030 to 2050.

In terms of health impacts, the research finds that from 2025 to 2050, the number of deaths caused by airport emissions under the four scenarios generally shows a slow upward trend. Among the four scenarios, the PSC-CCS pathway minimizes the health impacts of aviation emissions most effectively and the DAC-CCU scenario has the highest number of premature deaths. The CCU process, which involves the conversion and utilization of CO_2_, requires a large amount of reactants and generates additional greenhouse gas emissions and other pollutants during the reaction. In terms of health impacts, the CCS method performs better than the CCU method [62].

The number of deaths among men due to pollution exposure is higher than that among women. This phenomenon is primarily attributed to differences in physiological characteristics between the sexes, such as the larger lung capacity typically found in males, as well as differences in immune system responses, occupational exposures, and lifestyle factors. In addition to these reasons, factors such as working environments and job content also contribute to the higher mortality rate of lung diseases among men compared to women [63]. For instance, men are more likely to engage in frequent outdoor work and physical labor, thereby increasing their risk of developing lung diseases [64]. Specifically, the disease with the greatest gender difference in mortality is respiratory disease caused by SO_2_, and this disparity increases over time. This difference may be because men are more likely to smoke and have more comorbidities compared with women [65]. Besides, the sex differences in cardiovascular and cerebrovascular disease may be related to the cardioprotective effects of 17β-estradiol (E2) [66].

In 2030, the highest number of deaths occurs in the 80–84 age group and children aged 5–9 years have the lowest number of deaths. This trend is attributed to the fact that the elderly have weakened immune systems and a higher prevalence of chronic diseases, making them more susceptible to pollutants. In contrast, although children have immature immune systems and are more prone to acute diseases from short-term exposure, they experience less cumulative damage and thus have the lowest mortality rates. While in 2040 and 2050, the 85–89 age group is the most affected. This shift is likely due to improvements in medical care and increased life expectancy, which have led to a significant increase in the population base of the 85-89 years age group. There is also a big mortality disparity in cardiovascular diseases between the elderly and other age groups. The distinct time-activity patterns across different age groups may be the key attributes of this phenomenon [67].

Among the ten diseases related to aviation pollution, CCVD-SO_2_, RD-SO_2_, RD-NO_2_, and CCVD-CO have the highest fatality rates. NO_2_ and SO_2_ irritate the respiratory mucosa, increasing the risk of respiratory diseases [68,69]. Additionally, exposure to SO_2_ pollution has also been shown to impair cardiac function [70]. Conversely, the six diseases caused by PM_2.5_ account for the smallest proportion of deaths.

Furthermore, among the 1134 airports worldwide, those with the highest mortality rates include Chhatrapati Shivaji Maharaj International Airport, Paris Charles de Gaulle Airport, Netaji Subhas Chandra Bose International Airport, Ninoy Aquino International Airport, Incheon International Airport, Josep Tarradellas Barcelona-El Prat Airport, Soekarno-Hatta International Airport, Athens Eleftherios Venizelos Airport, Indira Gandhi International Airport, Cairo International Airport, and John F. Kennedy International Airport. First, the death toll near the airport is related to its geographical features. Chhatrapati Shivaji Maharaj International Airport, which ranks first in terms of the number of deaths, is located along the Arabian Sea coast. The dominant wind directions are southwest and northeast. The southwest monsoon can blow the emissions from the airport into the city, resulting in the accumulation of pollution and increasing deaths. Similarly, Paris Charles de Gaulle Airport, whose main wind direction is southwest, is also facing a similar situation, especially in the winter when pollution retention is severe. Moreover, existing studies have shown that dry, hot weather and warm, humid weather can increase respiratory mortality. Dry and hot weather can also enhance the association between SO_2_ and the mortality rate of cardiovascular and cerebrovascular diseases [71]. India has a tropical climate, and the high death toll near airports is also related to this factor. Secondly, these airports are all important transportation hubs. Paris Charles de Gaulle Airport had 460,916 aircraft take-offs and landings in 2024. The passenger flow was 70,290,260 person-times [72]. In 2024, Ninoy Aquino International Airport recorded a total passenger throughput of 50,356,465 people, and the aircraft took off and landed 293,433 times [73]. Incheon International Airport had 413,200 aircraft sorties, and the passenger flow was 71,156,947 person-times [74]. In contrast, Brussels National Airport, which reported lower mortality, recorded 23.61 million passengers and only 198,617 aircraft movements in 2024 [75]. The comparative analysis thus suggests that high-frequency flight operations inevitably exacerbate adverse health outcomes for residents near airports. The aircraft types and routes operated by different airports can also affect the death toll. Chhatrapati Shivaji Maharaj International Airport has a large number of old narrow-body aircraft, such as A320ceo and B737-800, taking off and landing here every day. There are several low-cost airlines that maintain high turnover. Similarly, airlines that frequently take off and land at Paris Charles de Gaulle Airport, such as Air France, operate a large number of long-range wide-body aircraft, such as A350 and B777, which emit higher levels of pollutants. Finally, the level of economic development, the use of green energy, and even the level of medical care can also affect the number of deaths near the airport. In a nutshell, most of these airports are located near coastal areas with warm and humid climates, where prevailing winds carry pollutants into densely populated residential zones. In addition, these airports experience high frequencies of take-offs and landings, and the aircraft models used tend to produce significant pollutant emissions.

Regarding economic performance, the findings indicate that all four carbon capture pathways—DAC-CCS, DAC-CCU, PSC-CCS, and PSC-CCU—generally exhibit a pattern where costs grow faster than revenues over time. For DAC pathways, although DAC-CCU yields higher revenues, its costs grow more rapidly, particularly after 2040, leading to a widening gap. This is primarily due to the high energy intensity of DAC processes and the additional feedstock requirements of DAC-CCU, particularly large volumes of hydrogen. These factors, combined with system complexity, result in significantly higher energy consumption than DAC-CCS. Furthermore, the DAC-CCU pathway is more technologically complex and has a less mature industrial value chain. Its heavy reliance on raw materials, high energy consumption, complex systems, and low industrial maturity contribute to elevated unit product costs, limiting large-scale deployment in the short term. Currently, DAC-CCS is relatively mature and more economically viable, whereas DAC-CCU remains in its early stages, resulting in a higher cost per unit of output. Therefore, in the selection of DAC-based strategies, DAC-CCS is the more cost-effective option compared to DAC-CCU. While DAC-CCS still yields negative returns, its fluctuations are smaller, indicating potential for optimization. Among PSC pathways, PSC-CCS demonstrates stronger net benefit stability. Although both its costs and revenues increase over time, the overall losses remain relatively steady, suggesting good medium- and long-term viability. In contrast, PSC-CCU experiences significantly higher cost growth compared to revenue growth, leading to expanding negative returns, particularly from 2040 to 2050. PSC features high capture efficiency, and in the CCS vs. CCU comparison, CCS involves simpler storage preparation, while CCU requires additional purification steps and may incur extra transportation costs post-capture, further widening the cost gap. From an industry perspective, PSC-CCS aligns more closely with existing policy frameworks and can be standardized across sectors like steel and cement. In contrast, PSC-CCU often demands more complex, case-specific technological routes and lacks general applicability. Additionally, PSC-CCS is more compatible with existing energy infrastructure, while PSC-CCU requires tailored carbon utilization scenarios. Without suitable downstream applications, conversion system costs rise steeply. PSC’s lower capture cost and higher technological maturity further exacerbate the disadvantages of CCU, reinforcing the stronger economic feasibility of PSC-CCS. PSC-CCS is more stable, conservative, and controllable, making it suitable for short- and medium-term deployment. Its cost-effectiveness is expected to improve further with rising carbon prices and better-developed storage infrastructure. Although PSC-CCU has higher revenue potential, its high costs render it unprofitable in the short term. Therefore, in the context of PSC-based pathways, PSC-CCS outperforms PSC-CCU in terms of net benefit performance and practical applicability.

A comparative evaluation of the four pathways indicates that PSC technologies offer greater economic feasibility than DAC technologies, with consistently lower associated costs. This advantage primarily stems from the nature of PSC as a point-source capture method, which requires less energy input compared to DAC. DAC involves capturing CO_2_ from ambient air, demanding large amounts of heat or electricity, thereby resulting in substantially higher energy consumption. Additionally, PSC handles smaller volumes of gas per unit of CO_2_ captured, contributing to lower operational intensity. PSC can also leverage existing industrial facilities and infrastructure, allowing for modular and scalable implementation. In contrast, DAC requires the construction of dedicated infrastructure, leading to longer construction timelines and higher upfront capital investment. In terms of technological maturity, PSC is more developed and stable, while DAC remains relatively nascent, with higher associated risks and uncertainties. These factors make both PSC-based pathways more favorable than their DAC-based counterparts.

When comparing CCU and CCS, CCS consistently demonstrates better cost-effectiveness. CCU involves additional utilization and conversion steps, requiring more complex equipment and higher energy consumption. Moreover, CCU heavily depends on market demand for CO_2_-derived products, introducing greater economic volatility. CCS, on the other hand, offers direct and permanent storage, reducing exposure to market fluctuations. From a cost-structure perspective, CCS primarily involves a one-time infrastructure investment, whereas CCU demands continuous operational input, making its lifecycle costs more variable and harder to predict. The fundamental difference lies in the post-capture phase: CCS is more predictable, lower in cost, and involves fewer uncertainties, resulting in consistently lower costs under the same capture technology.

In summary, PSC pathways are more economical than DAC pathways, and CCS options are more cost-effective than CCU. Combining these insights, PSC-CCS emerges as the most economically viable option, followed by DAC-CCS. PSC-CCU ranks third, while DAC-CCU is the least cost-effective, burdened by the highest costs and lowest net benefit performance among the four pathways.

Furthermore, there is consistency between PSC-CCS, especially the CCS, and the present policy framework. The International Energy Agency (IEA) has highlighted in its reports that fewer than 20 countries have so far established CCUS policies extending beyond research and development initiatives. The agency emphasizes that building a commercial market for CCUS requires a comprehensive suite of policy instruments and has called for enhanced international coordination to accelerate market development [76]. Consequently, understanding and analyzing policy frameworks across different regions is quite important. The European Commission attaches great importance to the development of CCUS, especially CCS. In November 2021, the commission released the fifth list of Projects of Common Interest (PCI) under the Trans-European Networks for Energy (TEN-E) Regulation, which includes six cross-border CO_2_ infrastructure projects with a focus on developing CO_2_ hubs. In February 2024, the commission further issued the Industrial Carbon Management Strategy, underscoring the need to boost the competitiveness of CO_2_ markets and transport infrastructure through legislative measures [77]. In the United States, incentives such as the 45Q tax credit serve as a primary mechanism, providing clear financial rewards for capturing, sequestering, or utilizing CO_2_. Notably, the credit offers a higher value for CO_2_ stored via CCS [78].

Lastly, the four technological pathways analyzed in this study are primarily deployed on the ground or within industrial infrastructure rather than onboard aircraft or other aviation activities. Therefore, they do not directly impact aviation safety. However, the intrinsic safety of the technologies is still a crucial aspect that cannot be neglected. Existing research has established safety assessment indicator systems based on three dimensions: chemicals, processes, and equipment, to evaluate the risks and potential incident consequences of CCUS projects [79]. Furthermore, some scholars have employed methods such as the Analytic Hierarchy Process (AHP) and expert scoring to analyze CCUS, concluding that the overall risk of such projects is relatively low [80].

Based on these findings, we proposes the following policy recommendations. First, optimize the selection of CCUS technology pathways and prioritize the deployment of PSC-CCS, given its favorable environmental, health, and economic performance. Governments should establish national strategies to prioritize investment in pipeline infrastructure for transporting CO_2_ captured via PSC technology to suitable geological storage sites, thereby accelerating the deployment of PSC technology. Second, in response to the continuously rising costs across all four methods, integrated infrastructure should be developed in key regions to form large-scale support systems, thereby reducing unit costs. Finally, airports with high mortality rates should implement real-time monitoring and public health early warning systems, alongside developing green buffer zones to better protect vulnerable populations such as the elderly. In addition, health impact assessment outcomes should be incorporated as one of the key reference indicators in the approval process for airport construction or expansion, ensuring accountability for public health.

Despite the meaningful findings of this study, certain limitations still remain. First, the cost-benefit analysis did not incorporate the economic benefits of avoided mortality across the different technological pathways, potentially leading to an underestimation of total benefits. Additionally, equipment retrofitting and maintenance costs were not included; only the costs of implementing the various CCUS technologies in the aviation sector were considered. Second, potential differences in CO_2_ transport, storage, or utilization across different airports were not considered; instead, a uniform standard was applied. The analysis was based on relatively idealized assumptions, including the premise that CCUS technologies could cover all carbon emissions and energy usage within the aviation sector. Future research will further examine the costs and benefits of these technologies under more realistic conditions. Lastly, this study used mortality as the sole indicator for assessing health impacts to facilitate a clearer comparison of the differences across various CCUS scenarios. Future research will incorporate additional indicators such as morbidity and the proportion of affected populations.

## 5 Conclusions

This study analyzes the health impacts and net benefit performance of four technological pathways integrating DAC, PSC, and CCUS. GEMM was applied to estimate mortality among residents living within 20 km of 1134 global airports. The results indicate a gradual increase in deaths attributable to airport emissions across all scenarios, with the PSC-CCS pathway demonstrating the least health damage. Specifically, pollution-related mortality was higher in males than in females, and the most affected age group was 80–89 years. The leading causes of death were respiratory and cardiovascular diseases linked to SO_2_, respiratory diseases associated with NO_2_, and cardiovascular conditions related to CO. Chhatrapati Shivaji Maharaj International Airport, Paris Charles de Gaulle Airport, and Netaji Subhas Chandra Bose International Airport consistently exhibited the highest mortality rates across all four scenarios. In cost-benefit analysis, PSC-CCS emerges as the most economically viable and feasible pathway, establishing it as the most favorable pathway in terms of both health and economic outcomes.

## CRediT authorship contribution statement

**Qiang Cui:** Writing – review & editing, Writing – original draft, Methodology, Data curation, Conceptualization. **Ying Zhou:** Writing – original draft, Validation, Data curation. **Xing-yu Tang:** Writing – original draft, Data curation, Conceptualization. **Xu-jie Sun:** Writing – original draft, Data curation. **Yu-xin Zhang:** Investigation, Data curation. **Ye Li:** Writing – review & editing, Writing – original draft, Conceptualization.

## Declaration of competing interest

The authors declare no competing interests.
