# Supplementary material for: Point source capture with storage yields superior aviation health benefits over direct air capture
Source: Eco Environ Health. 2026 Mar 21;5(2):100235. doi: 10.1016/j.eehl.2026.100235 (PMC13088995; doi:10.1016/j.eehl.2026.100235)
Supplement: Multimedia component 1 [file mmc1.docx]

**Supplementary Materials for**

**Point source capture with storage yields superior aviation health benefits over direct air capture**

Qiang Cui^a^**^*^**, Ying Zhou^a^, Xing-yu Tang^a^, Xu-jie Sun^a^, Yu-xin Zhang^a^, Ye Li^b*^

a. School of Economics and Management, Southeast University, Nanjing 211189, China

b. School of Business Administration, Nanjing University of Finance and Economics, Nanjing 210023, China

* Corresponding authors. E-mail address: [cuiqiang@seu.edu.cn](mailto:cuiqiang@seu.edu.cn) (Qiang Cui); [happyxiaoye@yeah.net (Ye](mailto:happyxiaoye@yeah.net%20(Ye) Li)

Content

[S1 Introduction of the AIM model 2](#_Toc24489)

[S2 The Gaussian Diffusion Model and GEMM 2](#_Toc2222)

[S2.1 The Gaussian Diffusion Model 3](#_Toc350)

[S2.2 GEMM 3](#_Toc22720)

[S3 Various price forecasts used in cost-benefit analysis 4](#_Toc30773)

[S4 DAC technology path and cost (including CCS and CCU) 6](#_Toc11064)

[S5 DAC Emissions Calculation Process 7](#_Toc6016)

[S6 PSC technical path and cost (including CCS and CCU) 8](#_Toc22115)

[S7 PSC Emissions Calculation Process 9](#_Toc4980)

[S8 Cost Sensitivity Analysis—CCUS Technology Cost 9](#_Toc8258)

[S9 Sensitivity analysis of mortality prediction 12](#_Toc23112)

[References 16](#_Toc17817)

**S1 Introduction of the AIM model**

This study primarily draws on the results of AIM v11, which incorporates multiple aviation-related modules. Using 2015 data as a baseline, it simulates outcomes for four scenarios. The framework of the model is illustrated in Fig. S1.

Fig. S1 AIM framework (source: Lynnette M. D. AIM2015: Documentation. <https://www.atslab.org/wp-content/uploads/2019/12/AIM-2015-Documentation-v9-122019.pdf> (2025) )

It defines four key scenarios:

1. Ref_Mid: Assumes no major aviation policies or adoption of alternative fuels.
2. Pol_Mid: Simulates the impact of specific aviation policies, including CORSIA (expected to take effect in early 2023), the EU and UK ETS, and medium demand growth.
3. Pol_Low: Maintains the same policies as Pol_Mid but assumes a lower demand growth rate.
4. H2_Mid: Considers the adoption of hydrogen energy under medium demand growth and fluctuating oil prices.

**S2 The Gaussian Diffusion Model and GEMM**

Based on the emission data, this paper first uses the Gaussian diffusion model to calculate the average concentrations of PM_2.5_, CO, SO₂, and NO₂ caused by aircraft activities at each airport. Then, using relevant population and disease data, based on the GEMM, it is obtained that the impact of PM_2.5_, CO, SO₂, and NO₂ emitted by aircraft activities at 1134 airports around the world from 2025 to 2050 on the death number of residents within 20 km of the airport.

**S2.1 The Gaussian Diffusion Model**

This paper first obtains the take-off and landing numbers of each airport from 2025 to 2050 based on the calculation results of the AIM model. According to the proportion of take-off and landing numbers of each airport (if the proportion of take-off and landing numbers of each airport remains unchanged), the PM_2.5_, CO, SO₂, and NO₂ emission data calculated by the AIM model are distributed to each airport to obtain the total emissions of each airport from 2025 to 2050. Then, the emission flow rate is then averaged over time by dividing the total emissions by the number of days in the month, hours in a day, and seconds in an hour, resulting in values expressed in g/s. Finally, the Gaussian diffusion model is employed to calculate the monthly average concentrations of PM_2.5_, CO, SO₂, and NO₂ at each airport, expressed in μg/m³.

According to the emission flow, the average concentration value is calculated by the Gaussian diffusion model, which has been widely applied in calculating emission concentration^4,5^. The Gaussian diffusion model considers the influence of factors. The specific steps of the Gaussian diffusion model are as follows:

$$C=\frac{E}{2\pi\mu\delta_{y}\delta_{z}}\times\exp(\frac{-y^{2}}{2{\delta_{y}}^{2}})\times P$$

$$P=2\exp\left( \frac{-h^{2}}{2{\delta_{z}}^{2}} \right)+\exp\sum_{n=1}^{2} \left[ \frac{-\left( nl-h \right)^{2}}{2{\delta_{z}}^{2}} \right]+\exp\sum_{n=1}^{2} \left[ \frac{-\left( nl+h \right)^{2}}{2{\delta_{z}}^{2}} \right]$$

$C$ is the concentration (μg/m^3^), $E$ is the emission flow (g/s), $\mu$ is the wind speed (m/s). $\delta_{y}$, $\delta_{z}$ are the diffusion coefficient of the horizontal and vertical directions. $h$ is the effective height. $n$ is the number of smoke reflections, in this study, $h=2$ and $n=3$. $l$ is the mixing layer height (m). Generally, for the coastal airports, $l=900 m$, and for the other ones,$l=1100 m$. For the possible emission height that may have effects on humans $y$, this study selects $y=1$, $y=2$, and $y=3$, and then calculate the average value as the final concentration.

The average wind speed $\mu$ at each airport is shown in Dataset S1.

**S2.2 GEMM**

The Global Exposure Mortality Model (GEMM) consists of two steps: calculating relative risk (RR) and estimating premature deaths. RR is derived from comparing actual and threshold concentrations, while premature deaths are calculated using data on population size and baseline mortality rates. GEMM has been widely used in health impact assessments.

The GEMM predicts hazard ratios for relative risk (RR) in logarithmic form, extending beyond the observed exposure range, with changes decreasing as exposure increases. The equation is

$$RR=\left\{ \begin{aligned} 1 C_{g}<C_{0} \\ \exp\left\{ \theta\times\log\left( 1+\frac{C_{g}-C_{0}}{\alpha} \right)\times\left[ 1+\exp\left( -\frac{C_{g}-C_{0}-\mu}{\vartheta\times C_{g}} \right) \right] \right\} C_{g}\geq C_{0} \end{aligned} \right.$$

$C_{g}$ is the actual concentration (μg/m^3^), $C_{0}$ is the threshold concentration (μg/m^3^) ^6^. $\theta$, $\alpha$, $\mu$, and $\vartheta$ are the parameters that need to be set in advance. According to the results of parameter sensitivity analysis^7^, we set $\theta=0.143$, $\alpha=1.6$, $\mu=15.5$, and $\vartheta=36.8$.

For the number of premature deaths, the equation is

$$M=POP\times\left( \frac{RR-1}{RR} \right)\times BMR$$

$RR$ is the relative risk; $POP$ is the population; $BMR$ is the base mortality rate; $M$ is the number of premature deaths.

The number of deaths by age group, gender, and disease at each airport from 2025 to 2050 is shown in Dataset S4.

**S3 Various price forecasts used in cost-benefit analysis**

This study focuses on forecasting key prices, including those of aviation kerosene, hydrogen energy, sustainable aviation fuel (SAF), and electricity. Drawing on existing literature, historical prices for these energy sources from 1990 to 2023 were compiled^1^. Since the hydrogen energy used in aviation primarily consists of green hydrogen, this study specifically analyzes the price of green hydrogen. Electricity prices are averaged across major regions, including China, the United States, and Europe.

In addition to energy prices, carbon trading prices are also a critical factor. These are based on the pricing trends within the EU carbon trading system. Detailed price data can be found in Table S1.

Table S1 Price list of various types

| Year | Jet A-1 ($/barrel) | Carbon price ($/t) |
| --- | --- | --- |
| 1990 | 39 |  |
| 1991 | 38.9 |  |
| 1992 | 38 |  |
| 1993 | 37 |  |
| 1994 | 38 |  |
| 1995 | 30.2 |  |
| 1996 | 31 |  |
| 1997 | 31 |  |
| 1998 | 37 |  |
| 1999 | 36 |  |
| 2000 | 42 |  |
| 2001 | 46 |  |
| 2002 | 49 |  |
| 2003 | 54 |  |
| 2004 | 60 |  |
| 2005 | 99 | 7.78 |
| 2006 | 124 | 31.1 |
| 2007 | 142 | 0.23 |
| 2008 | 162 | 9.67 |
| 2009 | 92 | 8.54 |
| 2010 | 76 | 8.12 |
| 2011 | 96 | 7.86 |
| 2012 | 110 | 7.5 |
| 2013 | 118 | 6.06 |
| 2014 | 70 | 6.76 |
| 2015 | 66 | 7.69 |
| 2016 | 52 | 4.88 |
| 2017 | 76 | 6.24 |
| 2018 | 84 | 16.37 |
| 2019 | 89 | 24.51 |
| 2020 | 26 | 18.54 |
| 2021 | 79 | 49.78 |
| 2022 | 96 | 86.53 |
| 2023 | 115 | 95.2086 |

This paper predicts various prices from 2024 to 2050. First, for JET-A, this paper uses the formula of the AIM model to calculate^2^. That is,

*P_jet_* = 0.2062 + 0.793 × (*P_oil_*/*Base P_oil_*).

*P_jet_* is the price of JET-A, *P_oil_* is the price of crude oil, and *Base P_oil_* is the price of crude oil in 2015. Therefore, to predict the price of JET-A, we only need to get the predicted value of crude oil price. This paper collects the forecast results of crude oil price by the US Department of Energy and substitutes them into the above formula to get the price range of JET-A from 2024 to 2050, as shown in Table S2.

The forecast for carbon trading prices is also based on existing research^3^, with annual data projections provided in Table S2.

Table S2 Forecast values of various prices from 2024 to 2050

| Year | Jet A-1 ($/barrel) | | Carbon price ($/t) | |
| --- | --- | --- | --- | --- |
|  | Min | Max | Min | Max |
| 2024 | 97.231 | 98.781 | 95.94 | 97.24 |
| 2025 | 94.964 | 98.066 | 96.67 | 99.27 |
| 2026 | 92.698 | 97.350 | 97.41 | 101.30 |
| 2027 | 90.431 | 96.634 | 98.14 | 103.33 |
| 2028 | 88.165 | 95.919 | 98.87 | 105.36 |
| 2029 | 85.898 | 95.203 | 99.61 | 107.38 |
| 2030 | 83.632 | 94.487 | 100.34 | 109.41 |
| 2031 | 81.365 | 93.771 | 101.07 | 111.44 |
| 2032 | 79.099 | 93.056 | 101.81 | 113.47 |
| 2033 | 76.832 | 92.340 | 102.54 | 115.50 |
| 2034 | 74.566 | 91.624 | 103.27 | 117.53 |
| 2035 | 72.299 | 90.908 | 104.00 | 119.56 |
| 2036 | 70.033 | 90.193 | 104.74 | 121.59 |
| 2037 | 67.766 | 89.477 | 105.47 | 123.62 |
| 2038 | 65.500 | 88.761 | 106.20 | 125.65 |
| 2039 | 63.233 | 88.045 | 106.94 | 127.68 |
| 2040 | 60.967 | 87.330 | 107.67 | 129.71 |
| 2041 | 58.700 | 86.614 | 108.40 | 131.74 |
| 2042 | 56.434 | 85.898 | 109.14 | 133.77 |
| 2043 | 54.167 | 85.183 | 109.87 | 135.79 |
| 2044 | 51.901 | 84.467 | 110.60 | 137.82 |
| 2045 | 49.634 | 83.751 | 111.33 | 139.85 |
| 2046 | 47.368 | 83.035 | 112.07 | 141.88 |
| 2047 | 45.101 | 82.320 | 112.80 | 143.91 |
| 2048 | 42.835 | 81.604 | 113.53 | 145.94 |
| 2049 | 40.568 | 80.888 | 114.27 | 147.97 |
| 2050 | 38.302 | 80.172 | 115.00 | 150.00 |

**S4 DAC technology path and cost (including CCS and CCU)**

First, according to the literature^4^, the technical roadmap of DAC-CCS is shown in Fig. S2.


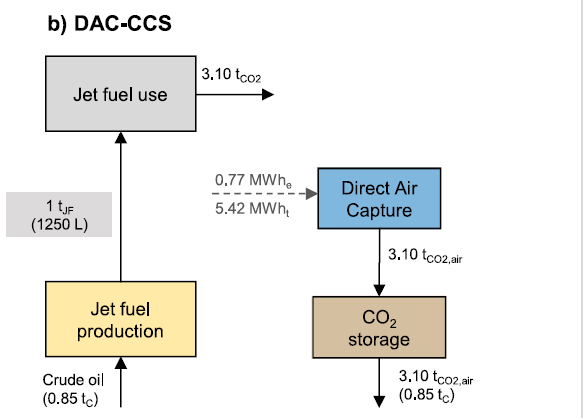


Fig. S2 The technical roadmap of DAC-CCS

As shown in Fig. S2, the basic process of DAC-CCS is to capture carbon dioxide, transport it through pipelines, and seal it. This paper assumes that there is no leakage during the transportation process.

According to the literature^4^, the technical roadmap of DAC-CCU is shown in Fig. S3.


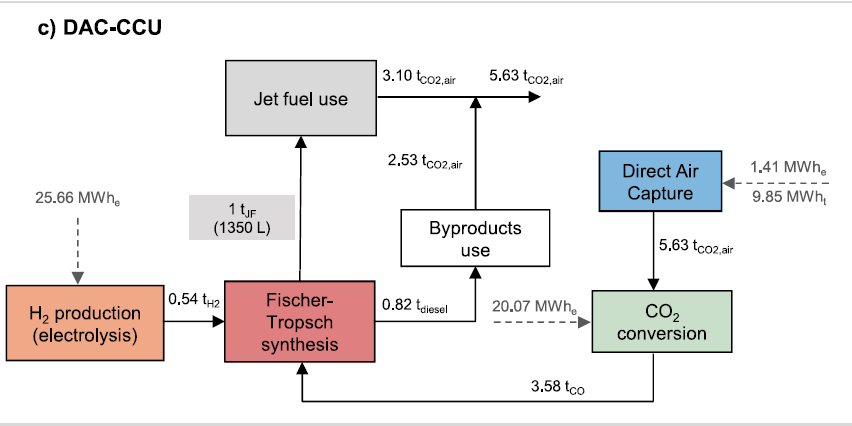


Fig. S3 The technical roadmap of DAC-CCU

As shown in Fig. S3, the basic process of DAC-CCU is to capture 5.63 t of carbon dioxide, transport it, and convert it into carbon monoxide. The carbon monoxide produced is Fischer-Tropsch synthesized with hydrogen produced by water electrolysis to produce one ton of aviation fuel and 0.82 t of diesel.

**S5 DAC Emissions Calculation Process**

The DAC process requires a certain amount of electricity, for example, the DAC-CCS process requires 2 kWh of electricity, while the DAC-CCU process requires 50 kWh of electricity. A DAC-CCS cycle’s emissions are shown in Table S3.

Table S3 The emissions of a DAC-CCS cycle

| Emissions | Amount (kg) |
| --- | --- |
| CO_2_ | 0.8664 |
| NO_x_ | 0.00235 |
| PM | 9.545e-04 |
| SO_2_ | 4.773e-06 |

The emissions of a DAC-CCU cycle (processing 5.63 t of carbon dioxide in total and producing 1 ton of JET-A) are shown in Table S4.

Table S4 The emissions of a DAC-CCU cycle

| Emissions | Amount (kg) |
| --- | --- |
| CO_2_ | 390.66 |
| CH_4_ | 61.5 |
| CO | 12.59 |
| NO_x_ | 70.707 |
| PM | 1.75086 |
| HC | 5.169 |
| SO_2_ | 1.193e-04 |

**S6 PSC technical path and cost (including CCS and CCU)**

First, according to the literature^4^, the technical roadmap of PSC-CCS is shown in Fig. S4.


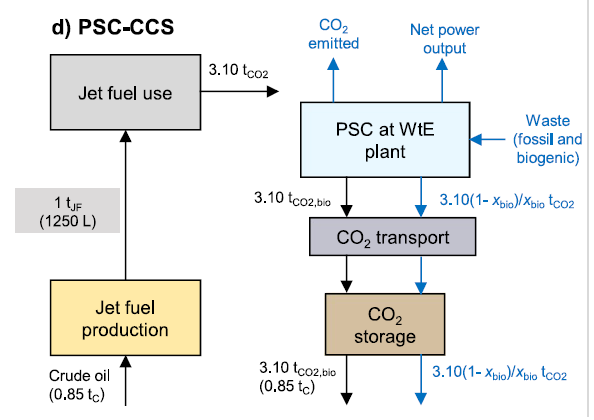


Fig. S4 The technical roadmap of PSC-CCS.

As shown in Fig. S4, the basic process of PSC-CCS is to capture carbon dioxide, transport it through pipelines, and seal it. This paper assumes that there is no leakage during the transportation process.

According to the literature^4^, the technical roadmap of PSC-CCU is shown in Fig. S5.


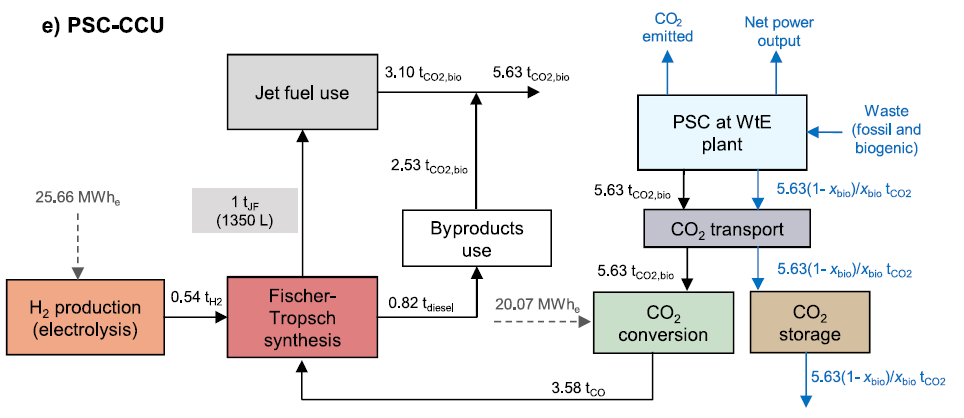


Fig. S5 The technical roadmap of PSC-CCU

As shown in Fig. S5, the basic process of PSC-CCU is to capture 5.63 t of carbon dioxide, transport it, and convert it into carbon monoxide. The carbon monoxide produced is Fischer-Tropsch synthesized with hydrogen produced by water electrolysis to produce one ton of aviation fuel and 0.82 t of diesel. This paper assumes that the electricity used in all processes is generated by clean energy.

According to the literature^4^, in 2020, the cost of PSC-CCS per ton of fuel is about US$856.65, while that of PSC-CCU is US$4283.25. According to its forecast, in 2050, the cost of PSC-CCS per ton of fuel will be about US$697, while that of PSC-CCU will be US$3252.88.

**S7 PSC Emissions Calculation Process**

PSC-CCU mainly involves three parts: production of Fischer-Tropsch fuel (diesel), emissions from burning Fischer-Tropsch fuel (diesel) and JET-A emissions. Therefore, the emissions generated by a PSC-CCU cycle (a total of 5.63 t of carbon dioxide are processed and 1 t of JET-A is produced) are shown in Table S5.

Table S5 Emissions from the PSC-CCU process per ton of fuel

| Emissions | Amount (kg) |
| --- | --- |
| CO_2_ | 369 |
| CH_4_ | 61.5 |
| CO | 12.59 |
| NO_x_ | 70.648 |
| PM | 1.727 |
| HC | 5.169 |

**S8 Cost Sensitivity Analysis—CCUS Technology Cost**

This paper conducts a sensitivity analysis on the technical costs of various CCUS scenarios, setting up four scenarios: existing cost +5%, existing cost +10%, existing cost -5%, and existing cost -10%. This is because the technical costs of CCUS only affect overall costs and not revenue. The results are shown in Tables S6-S9.

Table S6. DAC-CCS Cost Sensitivity Analysis (USD billion)

| Year | Original cost | +5% | +10% | -5% | -10% | Net benefit - min | Net benefit - max |
| --- | --- | --- | --- | --- | --- | --- | --- |
| 2025 | 0.00 | 0.00 | 0.00 | 0.00 | 0.00 | 0.00 | 0.00 |
| 2026 | 24.85 | 26.10 | 27.34 | 23.61 | 22.37 | 3.25 | 3.38 |
| 2027 | 48.65 | 51.08 | 53.51 | 46.21 | 43.78 | 6.56 | 6.90 |
| 2028 | 71.38 | 74.95 | 78.51 | 67.81 | 64.24 | 9.91 | 10.56 |
| 2029 | 93.04 | 97.70 | 102.35 | 88.39 | 83.74 | 13.31 | 14.35 |
| 2030 | 113.65 | 119.33 | 125.01 | 107.97 | 102.28 | 16.76 | 18.28 |
| 2031 | 133.19 | 139.85 | 146.51 | 126.53 | 119.87 | 20.26 | 22.34 |
| 2032 | 151.67 | 159.26 | 166.84 | 144.09 | 136.51 | 23.81 | 26.54 |
| 2033 | 169.09 | 177.55 | 186.00 | 160.64 | 152.18 | 27.40 | 30.87 |
| 2034 | 185.45 | 194.72 | 204.00 | 176.18 | 166.91 | 31.05 | 35.34 |
| 2035 | 200.75 | 210.78 | 220.82 | 190.71 | 180.67 | 34.74 | 39.94 |
| 2036 | 214.98 | 225.73 | 236.48 | 204.23 | 193.48 | 38.49 | 44.68 |
| 2037 | 228.15 | 239.56 | 250.96 | 216.74 | 205.33 | 42.28 | 49.56 |
| 2038 | 240.26 | 252.27 | 264.28 | 228.24 | 216.23 | 46.12 | 54.57 |
| 2039 | 251.30 | 263.87 | 276.43 | 238.74 | 226.17 | 50.01 | 59.71 |
| 2040 | 261.29 | 274.35 | 287.42 | 248.22 | 235.16 | 53.95 | 65.00 |
| 2041 | 270.21 | 283.72 | 297.23 | 256.70 | 243.19 | 57.94 | 70.41 |
| 2042 | 278.07 | 291.97 | 305.88 | 264.17 | 250.26 | 61.98 | 75.97 |
| 2043 | 284.87 | 299.11 | 313.35 | 270.62 | 256.38 | 66.07 | 81.66 |
| 2044 | 290.60 | 305.13 | 319.66 | 276.07 | 261.54 | 70.20 | 87.48 |
| 2045 | 295.28 | 310.04 | 324.80 | 280.51 | 265.75 | 74.39 | 93.44 |
| 2046 | 298.89 | 313.83 | 328.78 | 283.94 | 269.00 | 78.62 | 99.54 |
| 2047 | 301.44 | 316.51 | 331.58 | 286.36 | 271.29 | 82.90 | 105.77 |
| 2048 | 302.92 | 318.07 | 333.21 | 287.78 | 272.63 | 87.23 | 112.13 |
| 2049 | 303.35 | 318.51 | 333.68 | 288.18 | 273.01 | 91.61 | 118.64 |
| 2050 | 302.71 | 317.84 | 332.98 | 287.57 | 272.44 | 96.04 | 125.27 |

As shown in Table S6, even if the cost of DAC-CCS decreases by 10%, it is still far higher than its potential maximum benefit.

Table S7. DAC-CCU Cost Sensitivity Analysis (USD billion)

| Year | Original cost | +5% | +10% | -5% | -10% | Net benefit - min | Net benefit - max |
| --- | --- | --- | --- | --- | --- | --- | --- |
| 2025 | 0.00 | 0.00 | 0.00 | 0.00 | 0.00 | 0.00 | 0.00 |
| 2026 | 228.92 | 240.36 | 251.81 | 217.47 | 206.03 | 47.66 | 50.02 |
| 2027 | 450.07 | 472.57 | 495.08 | 427.57 | 405.06 | 93.21 | 99.50 |
| 2028 | 663.45 | 696.63 | 729.80 | 630.28 | 597.11 | 136.62 | 148.42 |
| 2029 | 869.07 | 912.52 | 955.98 | 825.62 | 782.16 | 177.92 | 196.79 |
| 2030 | 1066.92 | 1120.27 | 1173.61 | 1013.58 | 960.23 | 217.09 | 244.61 |
| 2031 | 1257.00 | 1319.86 | 1382.71 | 1194.15 | 1131.30 | 254.14 | 291.88 |
| 2032 | 1439.32 | 1511.29 | 1583.25 | 1367.36 | 1295.39 | 289.07 | 338.60 |
| 2033 | 1613.87 | 1694.57 | 1775.26 | 1533.18 | 1452.48 | 321.88 | 384.78 |
| 2034 | 1780.66 | 1869.69 | 1958.72 | 1691.62 | 1602.59 | 352.56 | 430.40 |
| 2035 | 1939.67 | 2036.66 | 2133.64 | 1842.69 | 1745.70 | 381.12 | 475.47 |
| 2036 | 2090.92 | 2195.47 | 2300.01 | 1986.38 | 1881.83 | 407.55 | 519.99 |
| 2037 | 2234.40 | 2346.12 | 2457.84 | 2122.68 | 2010.96 | 431.87 | 563.96 |
| 2038 | 2370.12 | 2488.63 | 2607.13 | 2251.61 | 2133.11 | 454.06 | 607.38 |
| 2039 | 2498.07 | 2622.97 | 2747.88 | 2373.17 | 2248.26 | 474.13 | 650.25 |
| 2040 | 2618.25 | 2749.16 | 2880.08 | 2487.34 | 2356.43 | 492.08 | 692.57 |
| 2041 | 2730.67 | 2867.20 | 3003.73 | 2594.13 | 2457.60 | 507.90 | 734.34 |
| 2042 | 2835.32 | 2977.08 | 3118.85 | 2693.55 | 2551.78 | 521.60 | 775.56 |
| 2043 | 2932.20 | 3078.81 | 3225.42 | 2785.59 | 2638.98 | 533.18 | 816.23 |
| 2044 | 3021.31 | 3172.38 | 3323.44 | 2870.25 | 2719.18 | 542.63 | 856.34 |
| 2045 | 3102.66 | 3257.79 | 3412.93 | 2947.53 | 2792.40 | 549.97 | 895.91 |
| 2046 | 3176.24 | 3335.06 | 3493.87 | 3017.43 | 2858.62 | 555.18 | 934.93 |
| 2047 | 3242.06 | 3404.16 | 3566.26 | 3079.95 | 2917.85 | 558.26 | 973.40 |
| 2048 | 3300.11 | 3465.11 | 3630.12 | 3135.10 | 2970.09 | 559.23 | 1011.32 |
| 2049 | 3350.39 | 3517.91 | 3685.42 | 3182.87 | 3015.35 | 558.07 | 1048.69 |
| 2050 | 3392.90 | 3562.55 | 3732.19 | 3223.26 | 3053.61 | 554.79 | 1085.50 |

As shown in Table S7, even with a 10% decrease in DAC-CCU costs, the potential benefits are still far higher than the maximum possible benefits.

Table S8. PSC-CCS Cost Sensitivity Analysis (USD billion)

| Year | Original cost | +5% | +10% | -5% | -10% | Net benefit - min | Net benefit - max |
| --- | --- | --- | --- | --- | --- | --- | --- |
| 2025 | 0.00 | 0.00 | 0.00 | 0.00 | 0.00 | 0.00 | 0.00 |
| 2026 | 8.73 | 9.16 | 9.60 | 8.29 | 7.85 | 3.25 | 3.38 |
| 2027 | 17.34 | 18.21 | 19.08 | 16.47 | 15.61 | 6.56 | 6.90 |
| 2028 | 25.84 | 27.14 | 28.43 | 24.55 | 23.26 | 9.91 | 10.56 |
| 2029 | 34.23 | 35.94 | 37.66 | 32.52 | 30.81 | 13.31 | 14.35 |
| 2030 | 42.51 | 44.64 | 46.76 | 40.38 | 38.26 | 16.76 | 18.28 |
| 2031 | 50.67 | 53.21 | 55.74 | 48.14 | 45.61 | 20.26 | 22.34 |
| 2032 | 58.73 | 61.66 | 64.60 | 55.79 | 52.85 | 23.81 | 26.54 |
| 2033 | 66.67 | 70.00 | 73.33 | 63.33 | 60.00 | 27.40 | 30.87 |
| 2034 | 74.49 | 78.22 | 81.94 | 70.77 | 67.04 | 31.05 | 35.34 |
| 2035 | 82.21 | 86.32 | 90.43 | 78.10 | 73.99 | 34.74 | 39.94 |
| 2036 | 89.81 | 94.30 | 98.79 | 85.32 | 80.83 | 38.49 | 44.68 |
| 2037 | 97.30 | 102.16 | 107.03 | 92.43 | 87.57 | 42.28 | 49.56 |
| 2038 | 104.67 | 109.91 | 115.14 | 99.44 | 94.21 | 46.12 | 54.57 |
| 2039 | 111.94 | 117.53 | 123.13 | 106.34 | 100.74 | 50.01 | 59.71 |
| 2040 | 119.09 | 125.04 | 131.00 | 113.13 | 107.18 | 53.95 | 65.00 |
| 2041 | 126.13 | 132.43 | 138.74 | 119.82 | 113.51 | 57.94 | 70.41 |
| 2042 | 133.05 | 139.71 | 146.36 | 126.40 | 119.75 | 61.98 | 75.97 |
| 2043 | 139.87 | 146.86 | 153.85 | 132.87 | 125.88 | 66.07 | 81.66 |
| 2044 | 146.57 | 153.90 | 161.22 | 139.24 | 131.91 | 70.20 | 87.48 |
| 2045 | 153.16 | 160.81 | 168.47 | 145.50 | 137.84 | 74.39 | 93.44 |
| 2046 | 159.63 | 167.61 | 175.59 | 151.65 | 143.67 | 78.62 | 99.54 |
| 2047 | 165.99 | 174.29 | 182.59 | 157.70 | 149.40 | 82.90 | 105.77 |
| 2048 | 172.25 | 180.86 | 189.47 | 163.63 | 155.02 | 87.23 | 112.13 |
| 2049 | 178.38 | 187.30 | 196.22 | 169.46 | 160.55 | 91.61 | 118.64 |
| 2050 | 184.41 | 193.63 | 202.85 | 175.19 | 165.97 | 96.04 | 125.27 |

As shown in Table S8, even with a 10% decrease in PSC-CCS costs, the potential benefits are still far higher than the maximum possible benefits.

Table S9. PSC-CCU Cost Sensitivity Analysis (USD billion)

| Year | Original cost | +5% | +10% | -5% | -10% | Net benefit - min | Net benefit - max |
| --- | --- | --- | --- | --- | --- | --- | --- |
| 2025 | 0.00 | 0.00 | 0.00 | 0.00 | 0.00 | 0.00 | 0.00 |
| 2026 | 136.20 | 143.02 | 149.83 | 129.39 | 122.58 | 47.66 | 50.02 |
| 2027 | 270.11 | 283.62 | 297.13 | 256.61 | 243.10 | 93.21 | 99.50 |
| 2028 | 401.73 | 421.82 | 441.90 | 381.64 | 361.56 | 136.62 | 148.42 |
| 2029 | 531.05 | 557.60 | 584.16 | 504.50 | 477.95 | 177.92 | 196.79 |
| 2030 | 658.08 | 690.98 | 723.88 | 625.17 | 592.27 | 217.09 | 244.61 |
| 2031 | 782.81 | 821.95 | 861.09 | 743.67 | 704.53 | 254.14 | 291.88 |
| 2032 | 905.24 | 950.51 | 995.77 | 859.98 | 814.72 | 289.07 | 338.60 |
| 2033 | 1025.39 | 1076.65 | 1127.92 | 974.12 | 922.85 | 321.88 | 384.78 |
| 2034 | 1143.23 | 1200.39 | 1257.56 | 1086.07 | 1028.91 | 352.56 | 430.40 |
| 2035 | 1258.78 | 1321.72 | 1384.66 | 1195.84 | 1132.91 | 381.12 | 475.47 |
| 2036 | 1372.04 | 1440.64 | 1509.25 | 1303.44 | 1234.84 | 407.55 | 519.99 |
| 2037 | 1483.00 | 1557.15 | 1631.30 | 1408.85 | 1334.70 | 431.87 | 563.96 |
| 2038 | 1591.67 | 1671.25 | 1750.84 | 1512.09 | 1432.50 | 454.06 | 607.38 |
| 2039 | 1698.04 | 1782.95 | 1867.85 | 1613.14 | 1528.24 | 474.13 | 650.25 |
| 2040 | 1802.12 | 1892.23 | 1982.33 | 1712.02 | 1621.91 | 492.08 | 692.57 |
| 2041 | 1903.91 | 1999.10 | 2094.30 | 1808.71 | 1713.52 | 507.90 | 734.34 |
| 2042 | 2003.39 | 2103.56 | 2203.73 | 1903.23 | 1803.06 | 521.60 | 775.56 |
| 2043 | 2100.59 | 2205.62 | 2310.65 | 1995.56 | 1890.53 | 533.18 | 816.23 |
| 2044 | 2195.49 | 2305.26 | 2415.04 | 2085.71 | 1975.94 | 542.63 | 856.34 |
| 2045 | 2288.09 | 2402.50 | 2516.90 | 2173.69 | 2059.28 | 549.97 | 895.91 |
| 2046 | 2378.40 | 2497.32 | 2616.24 | 2259.48 | 2140.56 | 555.18 | 934.93 |
| 2047 | 2466.42 | 2589.74 | 2713.06 | 2343.10 | 2219.78 | 558.26 | 973.40 |
| 2048 | 2552.14 | 2679.74 | 2807.35 | 2424.53 | 2296.92 | 559.23 | 1011.32 |
| 2049 | 2635.56 | 2767.34 | 2899.12 | 2503.78 | 2372.01 | 558.07 | 1048.69 |
| 2050 | 2716.69 | 2852.53 | 2988.36 | 2580.86 | 2445.02 | 554.79 | 1085.50 |

As shown in Table S9, even with a 10% decrease in the cost of PSC-CCS, its potential maximum benefit still far exceeds its actual cost.

Combined with Tables S6-S9, it is clear that if the cost trends of the four CCUS technology paths are the same, it will not change the key conclusions of this paper, thus verifying the robustness of the conclusions.

**S9 Sensitivity analysis of mortality prediction**

In this study, we conducted a sensitivity analysis of mortality prediction under four pathways. Based on the baseline prediction results, we further constructed four alternative scenarios: baseline +5%, baseline +10%, baseline -5%, and baseline -10%. As an illustrative example, we present the sensitivity analysis for male mortality from CO exposure under the PSC-CCS scenario. Fig. S6 reports the sensitivity results in 2030, 2040, and 2050. Tables S10-S12 provide the detailed numerical outcomes and the corresponding changes in the number of deaths across age groups associated with each 1% variation in the predicted mortality rate.

Overall, mortality outcomes across all age groups exhibit proportionally similar increases as the prediction deviation ranges from -10% to +10%. The five scenario curves maintain consistent shapes and display a linear increasing pattern, indicating strong robustness of our results. Even under the largest deviations (-10% and +10%), the differences from the baseline scenario remain within a reasonable range. Nevertheless, some heterogeneity exists across age groups. The youngest group (<15 years) shows the smallest variation. For instance, in 2030, a 1% change in the predicted mortality rate leads to only a 0.59 change in deaths among individuals aged 5-9 years. By contrast, the magnitude of variation increases substantially with age, peaking in the 80-89 years age group. This indicates markedly higher sensitivity among older populations, making them the dominant contributors to overall uncertainty in total mortality. Specifically, in 2050, each 1% change in the predicted mortality rate results in a change of 362.8069 deaths in the 85-89 years age group.


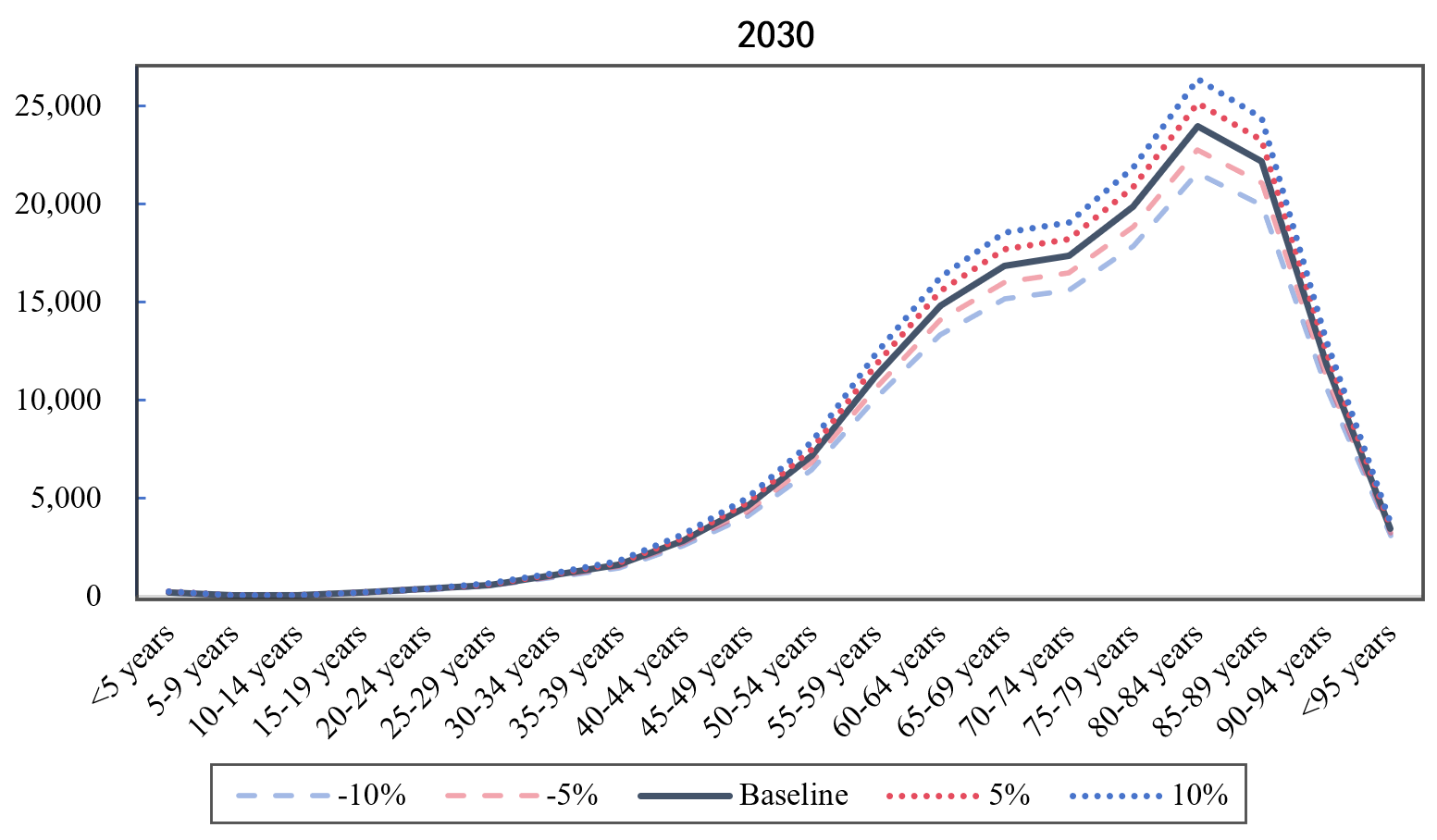


(a)


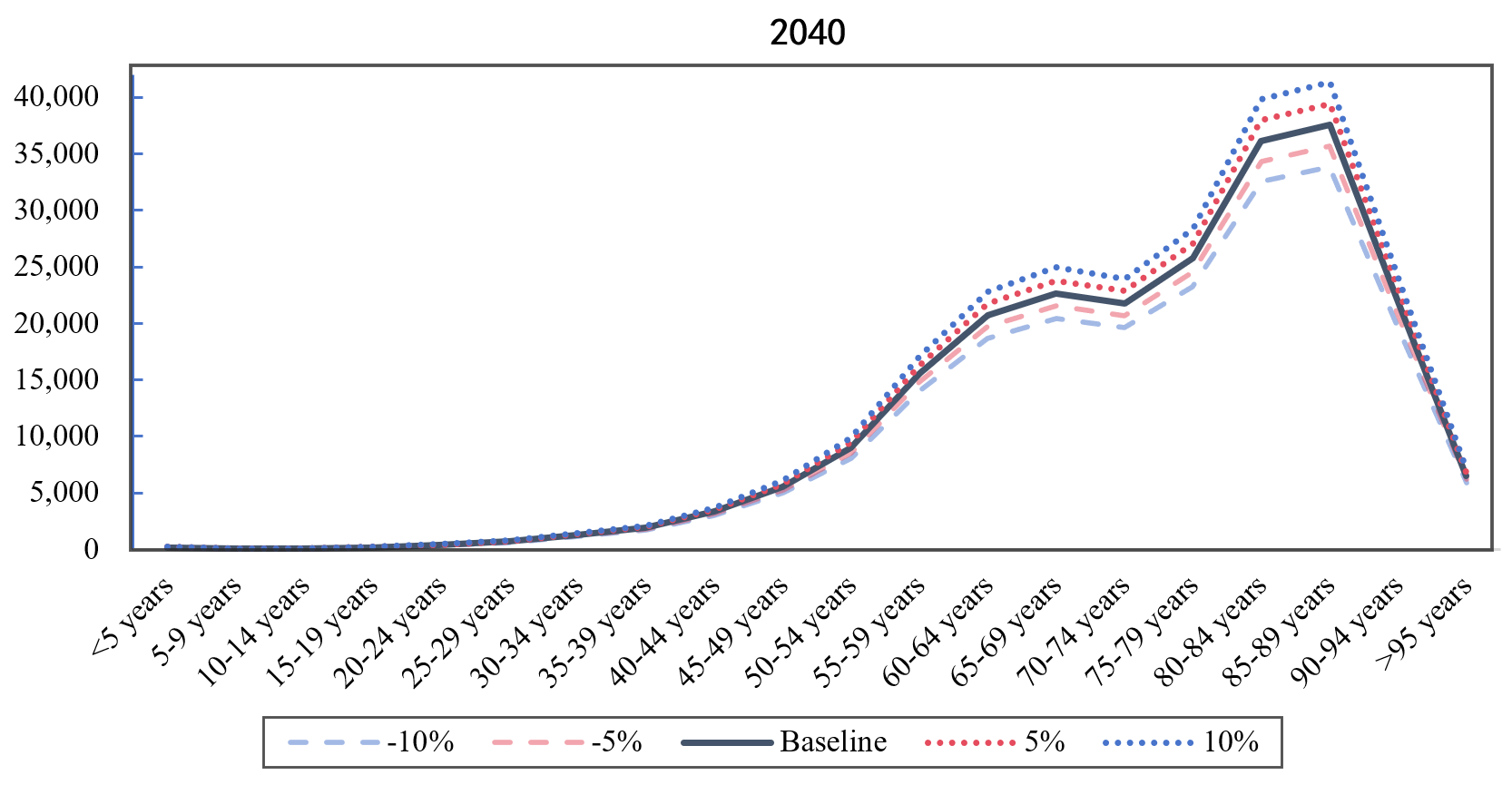


(b)


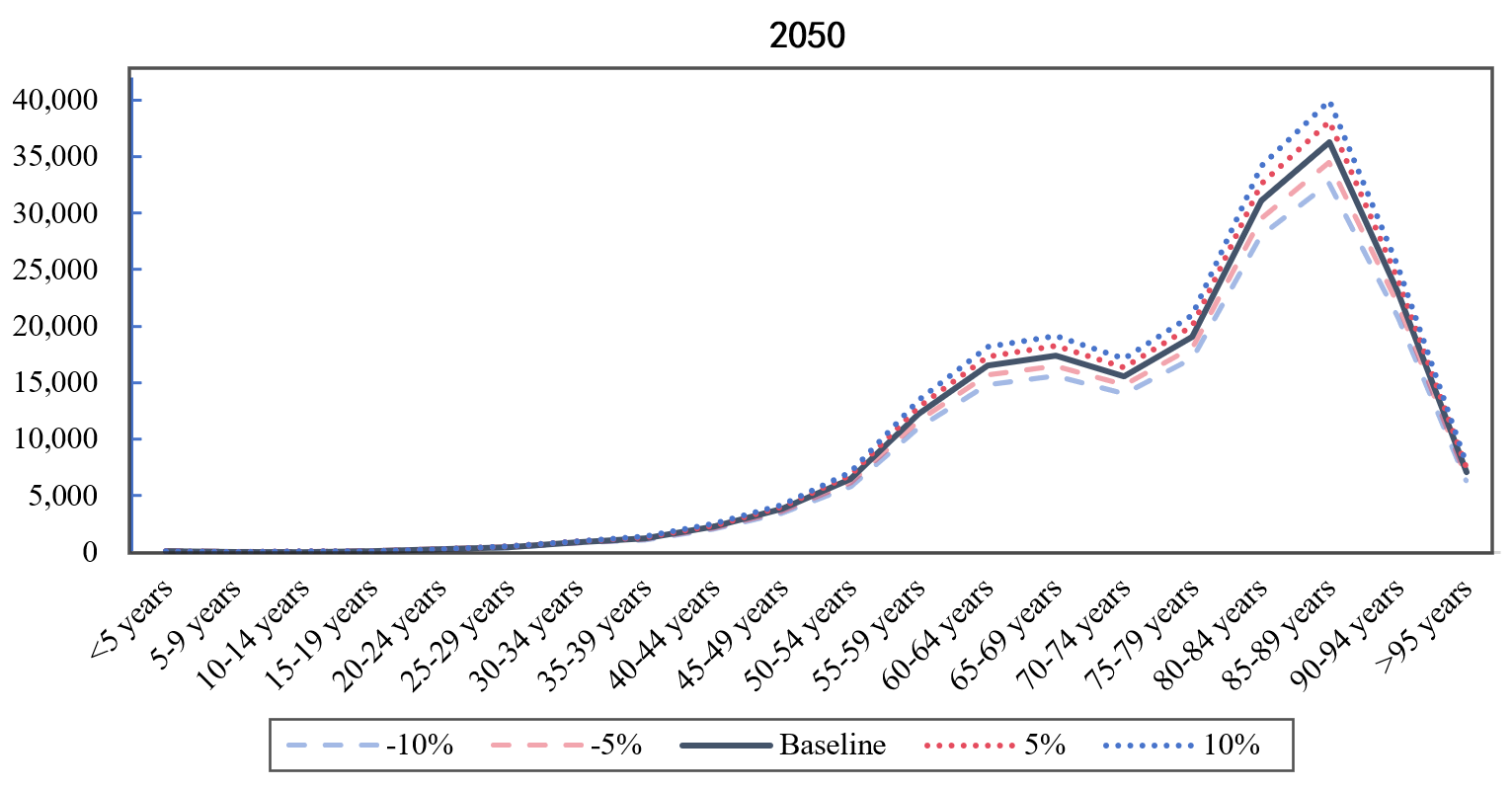


(c)

Fig. S6. Mortality prediction sensitivity analysis in 2030 (a), 2040 (b) and 2050 (c). Values are expressed in deaths.

Table S10. Mortality Prediction Sensitivity Analysis in 2030 (in deaths)

| Age | -10% | -5% | Baseline | 5% | 10% | Absolute Sensitivity |
| --- | --- | --- | --- | --- | --- | --- |
| <5 years | 195.88 | 206.76 | 217.65 | 228.53 | 239.41 | 2.18 |
| 5-9 years | 52.85 | 55.78 | 58.72 | 61.66 | 64.59 | 0.59 |
| 10-14 years | 57.66 | 60.86 | 64.07 | 67.27 | 70.47 | 0.64 |
| 15-19 years | 170.29 | 179.76 | 189.22 | 198.68 | 208.14 | 1.89 |
| 20-24 years | 333.22 | 351.73 | 370.24 | 388.75 | 407.27 | 3.70 |
| 25-29 years | 533.07 | 562.69 | 592.30 | 621.92 | 651.53 | 5.92 |
| 30-34 years | 976.11 | 1030.34 | 1084.57 | 1138.80 | 1193.03 | 10.85 |
| 35-39 years | 1473.30 | 1555.15 | 1637.00 | 1718.85 | 1800.70 | 16.37 |
| 40-44 years | 2576.95 | 2720.12 | 2863.28 | 3006.45 | 3149.61 | 28.63 |
| 45-49 years | 4107.18 | 4335.35 | 4563.53 | 4791.71 | 5019.88 | 45.64 |
| 50-54 years | 6445.10 | 6803.16 | 7161.22 | 7519.28 | 7877.34 | 71.61 |
| 55-59 years | 10102.60 | 10663.86 | 11225.11 | 11786.37 | 12347.63 | 112.25 |
| 60-64 years | 13329.92 | 14070.47 | 14811.02 | 15551.57 | 16292.13 | 148.11 |
| 65-69 years | 15153.14 | 15994.98 | 16836.82 | 17678.66 | 18520.50 | 168.37 |
| 70-74 years | 15610.04 | 16477.27 | 17344.49 | 18211.72 | 19078.94 | 173.44 |
| 75-79 years | 17858.24 | 18850.36 | 19842.48 | 20834.61 | 21826.73 | 198.42 |
| 80-84 years | 21553.53 | 22750.94 | 23948.36 | 25145.78 | 26343.20 | 239.48 |
| 85-89 years | 19931.19 | 21038.47 | 22145.76 | 23253.05 | 24360.34 | 221.46 |
| 90-94 years | 10667.03 | 11259.64 | 11852.25 | 12444.86 | 13037.48 | 118.52 |
| <95 years | 3090.17 | 3261.84 | 3433.52 | 3605.20 | 3776.87 | 34.34 |

Table S11. Mortality Prediction Sensitivity Analysis in 2040 (in deaths)

| Age | -10% | -5% | Baseline | 5% | 10% | Absolute Sensitivity |
| --- | --- | --- | --- | --- | --- | --- |
| <5 years | 179.7479 | 189.7339 | 199.7199 | 209.7059 | 219.6919 | 1.997199 |
| 5-9 years | 51.90361 | 54.78715 | 57.67068 | 60.55422 | 63.43775 | 0.576707 |
| 10-14 years | 58.34475 | 61.58613 | 64.8275 | 68.06888 | 71.31026 | 0.648275 |
| 15-19 years | 175.1398 | 184.8698 | 194.5998 | 204.3297 | 214.0597 | 1.945998 |
| 20-24 years | 378.4398 | 399.4642 | 420.4887 | 441.5131 | 462.5375 | 4.204887 |
| 25-29 years | 626.4948 | 661.3001 | 696.1053 | 730.9106 | 765.7159 | 6.961053 |
| 30-34 years | 1172.449 | 1237.585 | 1302.721 | 1367.857 | 1432.993 | 13.02721 |
| 35-39 years | 1708.212 | 1803.112 | 1898.013 | 1992.914 | 2087.814 | 18.98013 |
| 40-44 years | 3039.252 | 3208.099 | 3376.947 | 3545.794 | 3714.641 | 33.76947 |
| 45-49 years | 4987.457 | 5264.538 | 5541.619 | 5818.7 | 6095.781 | 55.41619 |
| 50-54 years | 8109.861 | 8560.409 | 9010.957 | 9461.505 | 9912.053 | 90.10957 |
| 55-59 years | 13997.99 | 14775.65 | 15553.32 | 16330.99 | 17108.65 | 155.5332 |
| 60-64 years | 18645.39 | 19681.25 | 20717.11 | 21752.96 | 22788.82 | 207.1711 |
| 65-69 years | 20401.53 | 21534.95 | 22668.36 | 23801.78 | 24935.2 | 226.6836 |
| 70-74 years | 19606.32 | 20695.56 | 21784.8 | 22874.04 | 23963.28 | 217.848 |
| 75-79 years | 23211.32 | 24500.84 | 25790.36 | 27079.88 | 28369.4 | 257.9036 |
| 80-84 years | 32555.99 | 34364.66 | 36173.32 | 37981.99 | 39790.65 | 361.7332 |
| 85-89 years | 33823.8 | 35702.9 | 37582 | 39461.09 | 41340.19 | 375.82 |
| 90-94 years | 19728.69 | 20824.73 | 21920.77 | 23016.81 | 24112.85 | 219.2077 |
| >95 years | 5879.107 | 6205.725 | 6532.342 | 6858.959 | 7185.576 | 65.32342 |

Table S12. Mortality Prediction Sensitivity Analysis in 2050 (in deaths)

| Age | -10% | -5% | Baseline | 5% | 10% | Absolute Sensitivity |
| --- | --- | --- | --- | --- | --- | --- |
| <5 years | 93.82977 | 99.04254 | 104.2553 | 109.4681 | 114.6808 | 1.042553 |
| 5-9 years | 28.99835 | 30.60936 | 32.22038 | 33.8314 | 35.44242 | 0.322204 |
| 10-14 years | 33.58505 | 35.45089 | 37.31673 | 39.18256 | 41.0484 | 0.373167 |
| 15-19 years | 102.4649 | 108.1574 | 113.8499 | 119.5424 | 125.2349 | 1.138499 |
| 20-24 years | 244.4966 | 258.0798 | 271.6629 | 285.2461 | 298.8292 | 2.716629 |
| 25-29 years | 418.8482 | 442.1176 | 465.3869 | 488.6563 | 511.9256 | 4.653869 |
| 30-34 years | 801.1134 | 845.6197 | 890.126 | 934.6323 | 979.1386 | 8.90126 |
| 35-39 years | 1126.674 | 1189.267 | 1251.86 | 1314.453 | 1377.046 | 12.5186 |
| 40-44 years | 2039.075 | 2152.357 | 2265.639 | 2378.921 | 2492.203 | 22.65639 |
| 45-49 years | 3445.256 | 3636.659 | 3828.062 | 4019.465 | 4210.868 | 38.28062 |
| 50-54 years | 5805.021 | 6127.522 | 6450.024 | 6772.525 | 7095.026 | 64.50024 |
| 55-59 years | 11033.28 | 11646.24 | 12259.19 | 12872.15 | 13485.11 | 122.5919 |
| 60-64 years | 14836.18 | 15660.41 | 16484.64 | 17308.88 | 18133.11 | 164.8464 |
| 65-69 years | 15625.33 | 16493.41 | 17361.48 | 18229.56 | 19097.63 | 173.6148 |
| 70-74 years | 14008.59 | 14786.85 | 15565.1 | 16343.36 | 17121.61 | 155.651 |
| 75-79 years | 17161.99 | 18115.44 | 19068.88 | 20022.32 | 20975.77 | 190.6888 |
| 80-84 years | 27973.7 | 29527.8 | 31081.89 | 32635.99 | 34190.08 | 310.8189 |
| 85-89 years | 32652.62 | 34466.65 | 36280.69 | 38094.72 | 39908.75 | 362.8069 |
| 90-94 years | 20756.77 | 21909.92 | 23063.08 | 24216.23 | 25369.38 | 230.6308 |
| >95 years | 6362.785 | 6716.273 | 7069.761 | 7423.249 | 7776.737 | 70.69761 |

**References**

1. IEA. Energy Prices. <https://www.iea.org/data-and-statistics/data-product/energy-prices> (2024)

2. Lynnette M. D. AIM2015: Documentation. <https://www.atslab.org/wp-content/uploads/2019/12/AIM-2015-Documentation-v9-122019.pdf> (2025).

3. Höglund-Isaksson L, Winiwarter W, Purohit P, et al. EU low carbon roadmap 2050: Potentials and costs for mitigation of non-CO2 greenhouse gas emissions. Energy Strategy Reviews, 1(2), 97-108 (2012).

4. Becattini V, Gabrielli P, Mazzotti M. Role of carbon capture, storage, and utilization to enable a net-zero-CO_2_-emissions aviation sector. Industrial & Engineering Chemistry Research, 60(18), 6848-6862 (2021).

5. Bofan Wang, Zhao Jia Ting, Ming Zhao. Sustainable aviation fuels: Key opportunities and challenges in lowering carbon emissions for aviation industry. Carbon Capture Science & Technology, 13, 100263(2024).

6. Sitanggang J W, Sunarsih E, Hasyim H. Literature review: analysis of exposure of vehicle emission gases (CO, NO2, SO2, PM2.5, and PM10) to public health risks. Journal of Social Research, 2(7), 2278-2287(2023).

7. Meo S A, Shaikh N, Alotaibi M. Association between air pollutants particulate matter (PM2.5, PM10), nitrogen dioxide (NO2), sulfur dioxide (SO2), volatile organic compounds (VOCs), ground-level ozone (O3) and hypertension. Journal of King Saud University - Science, 36(11), 103531(2024).
